# Prevalence and Determinants of Ideal Cardiovascular Health in Kenya: A Cross-Sectional Study Using Data From the 2015 Kenya STEPwise Survey

**DOI:** 10.5334/gh.1363

**Published:** 2024-10-23

**Authors:** James Odhiambo Oguta, Penny Breeze, Elvis Wambiya, Catherine Akoth, Grace Mbuthia, Peter Otieno, Oren Ombiro, Yvette Kisaka, Lilian Mbau, Elizabeth Onyango, Gladwell Gathecha, Pete R. J. Dodd

**Affiliations:** 1 Sheffield Centre for Health and Related Research, Division of Population Health, School of Medicine and Population Health, University of Sheffield. Sheffield, S1 4DA, United Kingdom; 2 School of Nursing, College of Health Sciences, Jomo Kenyatta University of Agriculture and Technology, P.O. Box 62000-0200 Nairobi, Kenya; 3 African Population and Health Research Center P.O. Box: 10787-00100, Nairobi, Kenya; 4 Medtronic LABS, Nairobi, Kenya; 5 Non-Communicable Diseases Division, Ministry of Health, Afya House, Cathedral Road, Nairobi, Kenya; 6 Kenya Cardiac Society, Nairobi, Kenya

**Keywords:** Ideal cardiovascular health, cardiovascular disease, prevalence, determinants, Kenya, cardiovascular disease risk factors

## Abstract

**Background:**

Kenya is experiencing a rising burden of cardiovascular diseases (CVDs) due to epidemiological and demographic shifts, along with increasing risk factors. Ideal cardiovascular health (iCVH), defined by the American Heart Association (AHA), encompasses eight metrics to evaluate cardiovascular well-being. This study assessed the prevalence and determinants of iCVH in Kenya.

**Methods:**

Data from the 2015 Kenya STEPwise survey on non-communicable disease risk factors, including 4,500 adults aged 18–69, were analysed. iCVH was assessed using 2022 AHA criteria based on seven factors: nicotine exposure, physical activity, diet, BMI, blood pressure, glucose, and lipid levels. A cardiovascular health (CVH) CVH score of ≥80% classified individuals as having iCVH. Multivariable binary and ordinal logistic regression identified factors associated with iCVH.

**Results:**

The mean CVH score in Kenya was 78.6% (95% CI: 77.9,79.2%), higher in females (79.3%), rural areas (79.5%), and non-drinkers (79.6%) than in males (77.9%), urban residents (77.0%), and alcohol drinkers (75.4%), respectively. The prevalence of iCVH (CVH score ≥80%) was 45.6%, while 6.4% had poor CVH (CVH score <50%). Only 1.2% achieved the maximum CVH score. iCVH prevalence declined with age and was lower among married individuals (43.7%), alcohol drinkers (32.3%), and urban residents (39.7%). Older adults had 50–80% lower odds of iCVH compared to those under 30 years. Alcohol users (AOR 0.5; p < 0.001) and urban residents (AOR 0.6; p < 0.001) were less likely to have iCVH. Residents of Nairobi and Central regions had 40–60% lower odds of iCVH compared to those in Rift Valley. The Kalenjin (AOR 0.5; p = 0.027) and Turkana (AOR 0.3; p = 0.002) ethnic groups had lower odds of iCVH compared to the Kisii.

**Conclusion:**

Less than half of Kenyan adults have iCVH, with poorer CVH status among older adults, urban residents, and alcohol users. Targeted public health interventions could mitigate the CVD burden and enhance health outcomes in Kenya.

## Introduction

The past three decades have seen the rise in the global burden of cardiovascular diseases (CVDs), characterised by the near doubling in prevalence, deaths and disability adjusted life years (DALYs) (1,2,3). In 2021, CVDs were the leading cause of all cause and non-communicable disease (NCD) related mortality and disability, with ischemic heart disease (IHD) and stroke causing the most impact (3). In sub-Saharan African (SSA), CVDs are responsible for more than one third of all NCD deaths (4). Kenya, a lower middle-income country in Eastern SSA, has also experienced an increasing burden of CVDs in the past decades. Estimates from the global burden of disease reveal that IHD and stroke are the leading causes of NCD-related deaths and fifth and seventh leading causes of all deaths in Kenya (2,5).

Behavioural and metabolic risk factors are associated with the increasing burden of CVDs. In Kenya, about 7.7% of the adult population is physically inactive (6), 13.5% smoke tobacco (7), 12.7% are heavy episodic alcohol drinkers (8), 18.3% report high dietary salt intake, and 13.7% take high sugar diet, while only 6% take the minimum required fruit and vegetable servings daily (9). Also, more than three-quarters of the Kenyan population possesses at least four of 12 different risk factors for NCDs, with 10% having more than seven risk factors (10). The upsurge in CVD results in significant health and economic impacts on the Kenyan health system and households.

The concept of “Ideal cardiovascular health (iCVH)” was introduced by the American Heart Association (AHA) in 2010 to aid the assessment of the cardiovascular health (CVH) status of the general population and improve the primordial and primary prevention of CVDs (11,12,13). Initially, iCVH was measured by seven CVH metrics (“Life’s Essential 7s”), which include three biological health factors (blood pressure (BP), cholesterol level, and blood sugar level) and four health behaviours (nicotine exposure, dietary intake, physical activity, and body mass index (BMI)) (11). Each CVH metric was defined by three categories (poor, intermediate, and ideal) and aggregated to generate a seven-point scale overall CVH index for an individual. In 2022, the AHA updated the definition of CVH to include sleep health (making “Life’s Essential 8s”) and proposed the use of a 100-point scale to assess each CVH metric, which would then be averaged to produce an overall CVH score, with individuals attaining at least 80% overall score defined as having an ideal (high) CVH status (13). Attaining iCVH metrics is associated with reduced risk of developing CVDs and improved overall health outcomes (14,15,16). A recent meta-analysis revealed that individuals with iCVH have about 40% and 82% reduced risk of atrial fibrillation and myocardial infarction, respectively, compared to those with poor CVH (17).

Previous studies assessing CVH status in SSA populations reveal that only about 0.3–3.3% of adults have all the seven CVH metrics at ideal levels (18,19,20,21). There exist gender differences in CVH metrics, with males more likely to have better CVH compared to females (22,23). Moreover, urban residence and advanced age have been associated with poorer CVH metrics (18,20,21,24). To better inform the scale-up of primordial and primary CVD prevention strategies in Kenya, it is important to understand the CVH status of the general population and the associated factors. Therefore, this study sought to assess the prevalence and factors associated with ideal CVH in Kenya using data from a nationally representative survey by applying the revised AHA guideline.

## Methods

### Study design and site

This is a cross-sectional study based on data from the World Health Organization (WHO) STEPS survey conducted in Kenya in 2015 (25). This was the first nationally representative survey that collected comprehensive information on NCD risk factors at household level for adults aged between 18 and 69 years in Kenya. Data were collected from all 47 counties in Kenya between April and June 2015 using WHO NCD risk factor surveillance questionnaires.

### Sampling and sample size

The sampling procedure for the STEPs survey has been described in detail elsewhere (25). In brief, a multistage sampling design was used to select a representative sample of adults aged 18–69 years using the fifth National Sample Surveys and Evaluation Programme (NASSEP V) as a sampling frame. Enumeration areas (EAs) obtained from the 2009 Kenya population and Housing census were used to develop the frame consisting of 5360 clusters which were split into four equal sub-samples. One randomly selected individual from an estimated sample size of 6000 households was targeted for the interviews after which 4500 adults aged 18–69 years were successfully interviewed.

### Measures

#### Outcome variable

The overall CVH score was defined by the cut-offs proposed by the revised AHA criteria based on four health behaviours (nicotine exposure, physical activity, diet, and BMI) and three health factors (blood pressure, glucose, and lipid levels) (13). Five CVH metrics (nicotine exposure, BMI, physical activity, blood pressure, and blood lipids) were constructed similarly to the AHA, whereas the other two (diet and blood glucose) were modified due to data inadequacies. The average daily fruit and vegetable serving was used to construct the scores for the diet metric due to lack of data on other components of Dietary Approaches to Stop Hypertension (DASH) diets. Similarly, a modified version of the diabetes status and blood glucose levels were used to define the blood glucose metric in the absence of glycated haemoglobin (HbA1c) variable within the dataset. Moreover, we did not include sleep health because it was not systematically collected in the dataset.

Table 1 presents the operational definition of the seven CVH metrics used to construct the outcome variable. Each participant was assigned a score of 0–100 against each of the seven CVH metrics. We then obtained a simple average of the seven CVH metrics contained in the dataset to generate a 100-point scale ordinal outcome variable (overall CVH score). Based on the new AHA recommendation (13), we categorised the overall CVH score into poor (0–49%), intermediate (50–79%) and high (≥80%) CVH. iCVH was defined as having an overall CVH score of at least 80% corresponding to the AHA definition of high CVH status (13). Individuals with prior history of CVD were classified as having poor CVH status.

**Table 1:** Scoring of CVH metrics using AHA criteria to estimate CVH status.


		AHA CRITERIA	THE STUDY’S OPERATIONAL DEFINITION

Diet	Measurement	Daily intake of a DASH-style eating pattern	Fruit and vegetable servings

	100	≥95th percentile	> =4.75 servings (≥95th percentile)

	80	75th–94th percentile	3.75–4.74 servings (75th–94th percentile)

	50	50th–74th percentile	2.5–3.74 servings (50th–74th percentile)

	25	25th–49th percentile	1.25–2.49 servings (25th–49th percentile)

	0	1st–24th percentile	<1.25 servings (1st–24th percentile)

Nicotine exposure	Measurement	Self-reported use of cigarettes or inhaled NDS	Self-reported use of tobacco (smoked or smokeless)

	100	Never Smoker	Never Smoker

	75	Former smoker, quit ≥5 y	Former smoker, quit ≥5 y

	50	Former smoker, quit 1–<5 y	Former smoker, quit 1–<5 y

	25	Former smoker, quit <1 y, or currently using inhaled NDS	Former smoker, quit <1 y

	0	Current smoker	Current Smoker

Physical activity (PA)	Measurement	Self-reported minutes of moderate or vigorous PA per week	Similar to AHA

	100	≥150	≥150

	90	120–149	120–149

	80	90–119	90–119

	60	60–89	60–89

	40	30–59	30–59

	20	1–29	1–29

	0	0	0

BMI	Measurement	Weight/(height squared-(kg/m^2^)	Weight/(height squared-(kg/m^2^)

	100	<25	<25

	70	25.0–29.9	25.0–29.9

	30	30.0–34.9	30.0–34.9

	15	35.0–39.9	35.0–39.9

	0	≥40.0	≥40.0

Blood Pressure	Measurement	Systolic and diastolic BPs (mm Hg)	Systolic and diastolic BPs (mm Hg)

	100	<120/<80 (optimal)	<120/<80 (optimal)

	75	120–129/<80 (elevated)	120–129/<80 (elevated)

	50	130–139 or 80–89 (stage 1 hypertension)	130–139 or 80–89 (stage 1 hypertension)

	25	140–159 or 90–99	140–159 or 90–99

	0	≥160 or ≥100	≥160 or ≥100

		If drug-treated level, subtract 20 points	If drug-treated level, subtract 20 points

Blood lipids	Measurement	Non–HDL cholesterol (mg/dL)	Non–HDL cholesterol (mmol/L)

	100	<130	<3.3

	60	130–159	3.3–4.0

	40	160–189	4.1–4.8

	20	190–219	4.9–5.6

	0	≥220	≥5.7

Blood Glucose	Measurement	FBG (mg/dL) or HbA1c (%)	FBG (mmol/l)

	100	No history of diabetes and FBG <100 (or HbA1c <5.7)	No diabetes and FBG <5.6 mmol/l

	60	No diabetes and FBG 100–125 (or HbA1c 5.7–6.4) (prediabetes)	No diabetes and FBG 5.6–6.9 mmol/l

	40	Diabetes with HbA1c <7.0	Diabetic and FBG <7.0 mmol/l

	30	Diabetes with HbA1c 7.0–7.9	

	20	Diabetes with HbA1c 8.0–8.9	Diabetic and FBG > = 7.0

	10	Diabetes with Hb A1c 9.0–9.9	

	0	Diabetes with HbA1c ≥10.0	


DASH-Dietary Approaches to stop hypertension; NDS-Nicotine Delivery System; AHA-American Heart Association; BP-Blood Pressure; HDL-High Density Lipoprotein; FBG-Fasting Blood Glucose; HbA1c-Hemoglobin A1c; BMI-Body Mass Index.

#### Explanatory variables

Sociodemographic factors included sex (male and female), marital status (in a union and not in a union), highest level of education (No formal education, primary education, and secondary and higher), age group (<30, 30–39, 40–49, and 50+), and occupation (salaried, self-employed, and unemployed/unpaid). Principal component analysis was used to construct household asset-based wealth index that was then categorised into five wealth quintiles (poorest-quintile 1, poorer-quintile 2, middle-quintile 3, richer-quintile 4, and richest-quintile 5) taking into account the clustered sampling design (26,27). Other independent variables included alcohol intake (never/past drinker and current user), place of residence (rural and urban), and region (Central, Eastern, Nyanza, Coast, Nairobi, Western, North-Eastern, and Rift Valley). Ethnicity was defined as Kikuyu, Embu, Kalenjin, Kamba, Borana, Kisii, Luhya, Luo, Maasai, Meru, Mijikenda, Somali, Turkana, and Other.

### Statistical Analysis

All the statistical analyses were performed using Stata version 18.0 (Stata Corporation, College Station, TX), while multiple imputation was performed in R Statistical Software (version 4.4.1). We adjusted for the clustered sampling design by using svy command in STATA, with the enumeration area being the primary sampling unit and individuals stratified by rural-urban residence considering the sampling weights used to select the study participants. Frequencies and percentages were used to summarise the sample characteristics. The chi-squared test of independence was used to assess the relationship between the outcome and explanatory variables. We used graphs to present the mean score for each of CVH metrics by sex. Using the revised WHO CVD risk equation for Eastern sub-Saharan Africa (SSA) (28), we predicted the 10-year CVD risk for individuals in the dataset. Both laboratory (lab-based) and non-laboratory (non-lab) based risk profiles were estimated. We used scatter plots and Pearson’s correlation coefficient to explore the relationship between the overall CVH score and the predicted 10-year CVD risk. To assess the factors associated with iCVH, we performed unadjusted and multivariable binary logistic regression analysis.

We identified the explanatory variables and confounders through the review of relevant literature (6,7,10,18,29,30) and addressed potential confounding by performing adjusted and stratified analyses (31). We assessed multicollinearity among the explanatory variables using variance inflation factor (VIF) and tolerance (defined as the reciprocal of VIF) levels (32). As a rule of the thumb, variables with VIF greater than 10 or tolerance less than 0.1 would warrant further investigation (32,33,34). None of the variables included in our model met the criteria for multicollinearity. All explanatory variables in the unadjusted analyses were included in the adjusted models. We reported unadjusted (crude) and adjusted odds ratios and assessed statistical significance at p-value ≤ 0.05.

The model goodness of fit was assessed using the Hosmer-Lemeshow test (35,36,37) and computation of the area (AUC) under the receiver operating characteristic (ROC) curve (38,39). We performed complete case analysis, the results of which are reported in the main text, and imputed missing data as a sensitivity analysis. To handle missing data, we first assessed the pattern of missingness and then performed multiple imputation using chained equations (MICE) (40,41,42). We assumed that data were missing at random and performed 80 imputations, which was informed by model convergence diagnostics (Supplementary Figures 1 and 2). Additional sensitivity analyses were performed by stratifying the analysis by residence, performing the adjusted analysis on the imputed dataset. Furthermore, a multivariable ordinal logistic regression model was performed using the overall CVH score as a sensitivity analysis. To test the robustness of the results, we performed Bonferroni correction for multiple comparisons to reduce the probability of type 1 error (43). This study was reported using STROBE guidelines for cross-sectional studies (Supplementary Table 4).

## Results

### Sample characteristics

The final sample for complete case analysis was 3818 adults, while the imputed analysis had 4500 adults. A higher proportion of participants in the final sample were females (59%), aged 18–29 (32.5%), in a marital union (67.9%), had no formal education (39.9%), unemployed (41.1%), non-drinkers of alcohol (79.2%), belonged to the Kalenjin (16.9%) and Kikuyu (16%) ethnic groups, resided in rural areas (52.1%), and in the Rift Valley region (31.1%). There was an almost even distribution of the sample across the five wealth quintiles (Table 2).

**Table 2:** Sample characteristics and prevalence of ideal CVH in Kenya.


VARIABLE	SAMPLE	PREVALENCE OF IDEAL CVH (OVERALL CVH SCORE > = 80%)						

		OVERALL (KENYA)			RURAL KENYA		URBAN KENYA	
			
	n (%)	n	% (95% CI)	p-value (Chi2)	n = 1988	p-value (Chi2)	n = 1830	p-value (Chi2)

**Total**	3818 (100)	1,674	45.6 [42.6,48.6]		48.7 [45.3, 52.1]		39.7 [35.0, 44.7]	

**Sex**								

Female	2250 (59)	1,022	47.4 [44.4,50.3]		49.5 [45.6,53.3]		43.0 [38.9,47.1]	

Male	1568 (41)	652	43.8 [39.5,48.3]	0.1224	47.8 [43.0,52.3]	0.5321	37.0 [30.5,44.1]	0.0837

**Age group (years)**								

18–29	1242 (32.5)	725	57.4 [52.3,62.3]		62.4 [57.5,67.0]		49.8 [41.5,58.2]	

30–39	1066 (27.9)	490	43.3 [38.5,48.3]		47.3 [41.5,53.2]		35.9 [28.5,44.1]	

40–49	697 (18.3)	249	34.0 [27.8,40.7]		41.2 [34.8,47.9]		20.3 [11.6,32.9]	

50+	813 (21.3)	210	25.6 [21.6,30.2]	<0.001	26.2 [21.7,31.3]	<0.001	23.9 [15.8,34.4]	<0.001

**Marital Status**								

In a union	252 (67.9)	1,134	43.7 [40.2,47.6]		48.4 [44.6,52.3]		34.5 [28.6,40.8]	

Not in a union	1226 (32.1)	540	48.8 [45.2,52.4]	0.0203	49.2 [45.0,53.3]	0.727	47.1 [41.0,53.4]	0.0018

**Education**								

No formal	1524 (39.9)	611	43.1 [38.7,47.6]		45.6 [40.8,50.5]		32.7 [24.4,42.2]	

Primary	1237 (32.4)	555	44.0 [39.3,48.8]		48.8 [43.6,54.0]		33.2 [26.2,41.1]	

Secondary +	1057 (27.7)	508	50.0 [44.2,55.7]	0.1059	54.5 [48.2,61.6]	0.072	46.3 [38.4,54.5]	0.021

**Occupation**								

Unemployed/Unpaid	1567 (41.1)	717	47.6 [43.1,50.9]		49.7 [45.1,54.4]		40.4 [30.8,50.7]	

Self-Employed	1536 (40.2)	671	46.6 [43.1,50.9]		47.5 [42.5,52.6]		44.7 [37.5,52.2]	

Employed/Salaried	715 (18.7)	286	39.9 [33.2,47.0]	0.1212	48.1 [40.8,55.6]	0.7798	32.7 [24.7,41.9]	0.1569

**Wealth quintile**								

Quintile 5	829 (21.7)	353	45.0 [39.2,51.0]		45.7 [39.2,52.3]		39.8 [29.9,50.7]	

Quintile 4	815 (21.4)	356	44.9 [40.1,49.9]		47.8 [42.2,53.4]		36.5 [28.5,45.5]	

Quintile 3	793 (20.8)	377	47.1 [40.9,53.5]		53.8 [48.5,58.9]		33.3 [23.1,45.4]	

Quintile 2	737 (19.3)	317	42.4 [36.2,48.9]		45.9 [39.3,52.7]		38.2 [28.2,49.4]	

Quintile 1	644 (16.9)	271	46.9 [40.3,53.7]	0.7904	52.8 [46.7,58.8]	0.1447	44.7 [36.1,53.6]	0.3955

**Alcohol intake**								

Never/Past drinker	3024 (79.2)	1419	49.6 [46.6,52.5]		52.5 [49.0,55.9]		44.2 [39.7,48.8]	

Current user	794 (20.8)	255	32.3 [26.6,38.4]	<0.001	34.9 [28.3,42.1]	<0.001	29.2 [20.7,39.4]	0.0023

**Residence**								

Rural	1988 (52.1)	918	48.7 [45.3,52.0]		–		–	

Urban	1830 (47.9)	756	39.7 [35.0,44.6]	0.0024	–		–	

**Region**								

Rift Valley	1189 (31.1)	535	47.0 [42.6,51.4]		45.7 [40.4, 51.2]		50.1 [43.4, 56.8]	

Eastern	695 (18.2)	252	39.2 [34.1,44.6]		41.6 [36.0, 47.4]		30.9 [24.9, 37.8]	

Nyanza	487 (12.8)	287	62.4 [52.7,71.2]		65.4 [52.8, 76.2]		54.4 [42.7, 65.7]	

Coast	419 (11)	165	41.2 [32.7,50.3]		41.4 [27.6, 56.7]		41.0 [31.8, 50.9]	

Nairobi	51 (1.3)	17	34.8 [26.8,43.8]		-		34.8 [26.7, 43.8]	

Western	368 (9.7)	169	51.4 [46.7,56.1]		52.7 [47.4, 57.9]		45.9 [38.9, 53.2]	

North Eastern	177 (4.6)	77	49.5 [42.6,56.6]		52.7 [44.5, 60.7]		32.6 [24.1, 42.4]	

Central	432 (11.3)	172	38.0 [29.9,46.9]	<0.001	44.1 [37.3, 51.1]	0.0019	29.7 [18.7, 43.7]	0.003

**Ethnicity**								

Kisii	195 (5.1)	119	62.9 [49.3,74.8]		72.0 [58.9, 82.3]		50.9 [30.3, 71.2]	

Embu	86 (2.3)	34	41.2 [23.3,61.8]		43.3 [23.9, 65.1]		37.8 [9.9, 77.0]	

Kalenjin	644 (16.9)	299	42.7 [36.7,48.9]		46.3 [39.6, 53.1]		35.6 [25.2, 47.6]	

Kamba	347 (9.1)	120	33.5 [28.1,39.3]		35.6 [28.8, 42.9]		31.0 [23.5, 39.7]	

Borana	18 (0.5)	7	33.5 [12.3,64.3]		47.3 [41.1, 53.4]		33.5 [12.3, 64.5]	

Kikuyu	612 (16)	258	45.5 [40.2,51.0]		49.0 [43.7, 54.4]		43.8 [35.3, 52.6]	

Luhya	476 (12.5)	200	43.9 [37.2,50.9]		56.5 [42.5, 69.5]		34.8 [26.0, 44.9]	

Luo	414 (10.8)	224	53.7 [44.7,62.5]		54.3 [42.0, 66.1]		50.7 [39.7, 61.5]	

Maasai	60 (1.6)	27	52.9 [40.7,64.7]		46.5 [38.6, 54.6]		40.0 [10.3, 79.5]	

Meru	221 (5.8)	88	43.3 [35.9,51.2]		41.6 [24.3, 61.3]		26.0 [14.9, 41.3]	

Mijikenda	143 (3.8)	53	37.7 [24.8,52.6]		51.8 [43.8, 59.7]		28.8 [17.9, 42.9]	

Somali	186 (4.9)	79	47.5 [40.2,55.0]		25.4 [14.9, 39.8]		27.8 [16.2, 43.4]	

Turkana	82 (2.2)	24	26.2 [17.2,37.9]		55.1 [38.0, 71.1]		30.1 [21.7, 40.1]	

Other	334 (8.8)	142	50.8 [38.4,63.1]	0.0013	48.7 [45.3, 52.1]	0.0034	43.3 [27.3, 60.9]	0.2455


### Prevalence of ideal CVH in Kenya

#### Overall ideal CVH Prevalence

The overall prevalence of ideal CVH in Kenya (CVH score ≥ 80%) was 45.6% (1674/3818; 95% CI 42.6, 48.6) while 6.4% (95% CI; 5.0,8.2%) had poor CVH status. However, only 1.2% (95% CI; 0.7–2.0%) of Kenyan adults had an overall CVH score of 100% (all the seven CVH metrics at maximum score). The prevalence of iCVH decreased by age and was lower among the married (43.7% vs. 48.8%), alcohol drinkers (32.3% vs. 49.6%), and urban residents (39.7% vs. 48.7%). Alcohol users, the married, and urban residents had significantly lower prevalence of iCVH metrics compared to non-users, the single, and rural residents, respectively. There was a significant difference in the prevalence of iCVH by region (p < 0.001) and ethnicity (p = 0.002). The Nyanza region (62.4%) had the highest prevalence of iCVH while Nairobi (34.8%) had the lowest. The Kisii (62.9%) ethnic tribe had the highest iCVH prevalence, while the Turkana (26.2%) had the lowest (Table 2).

### Mean overall CVH score

**Figure 1:**
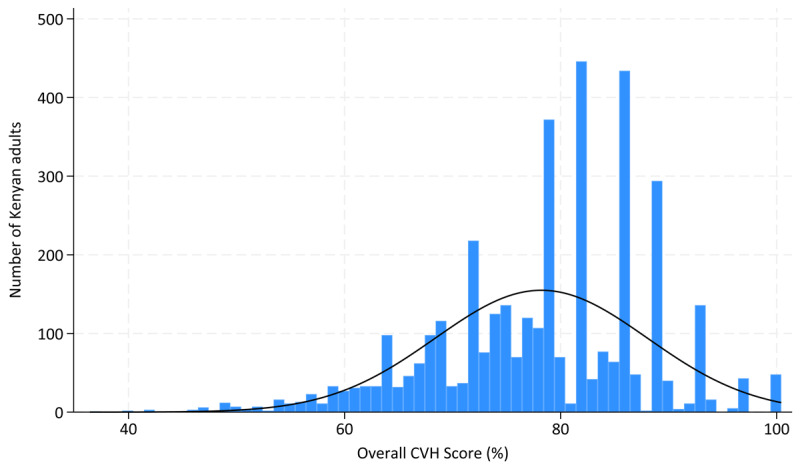
Distribution of overall CVH score.

**Figure 2:**
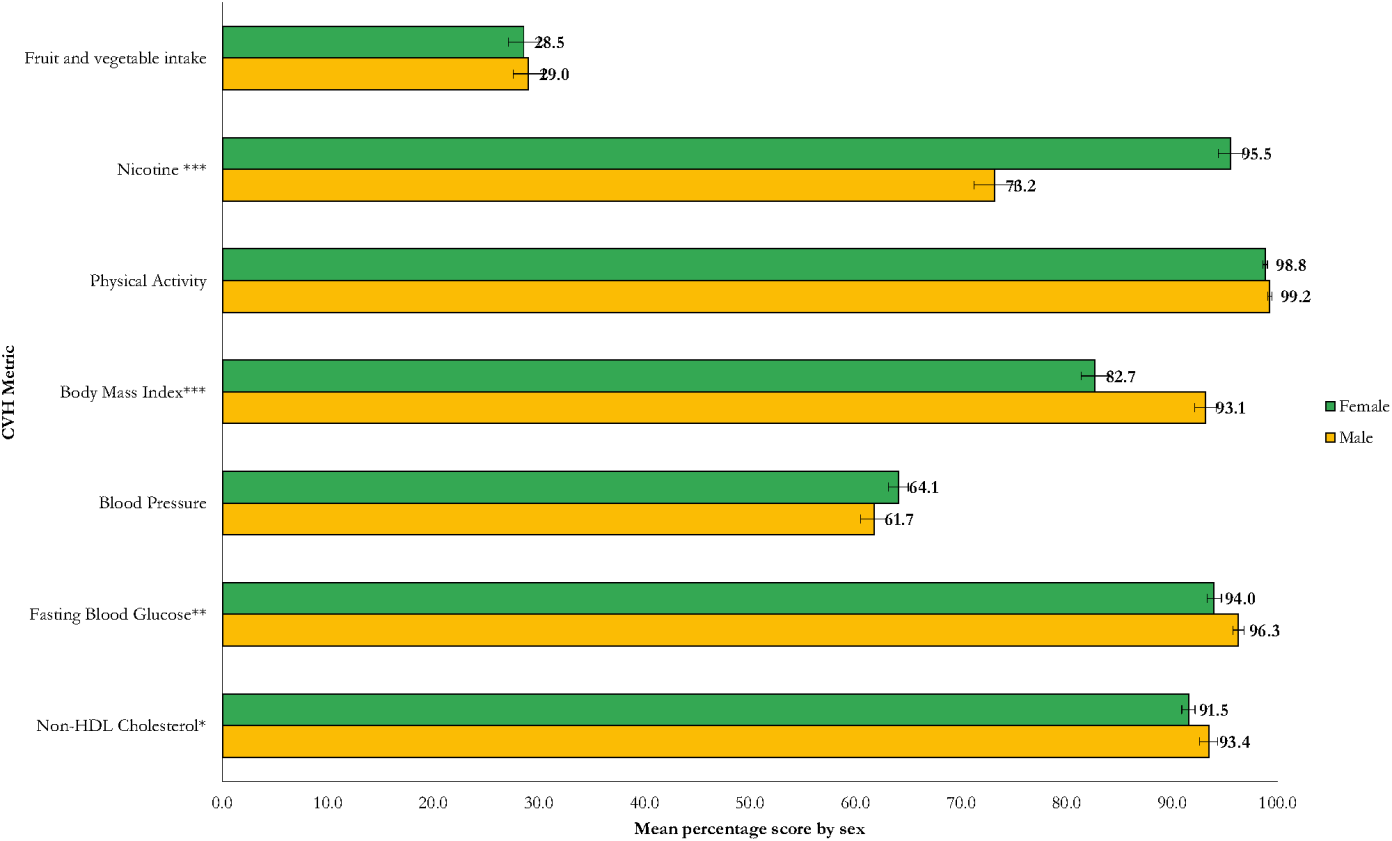
Distribution of CVH metrics by sex (Statistically significant difference at p < 0.001 ***, p < 0.01**, p < 0.05*).

**Figure 3:**
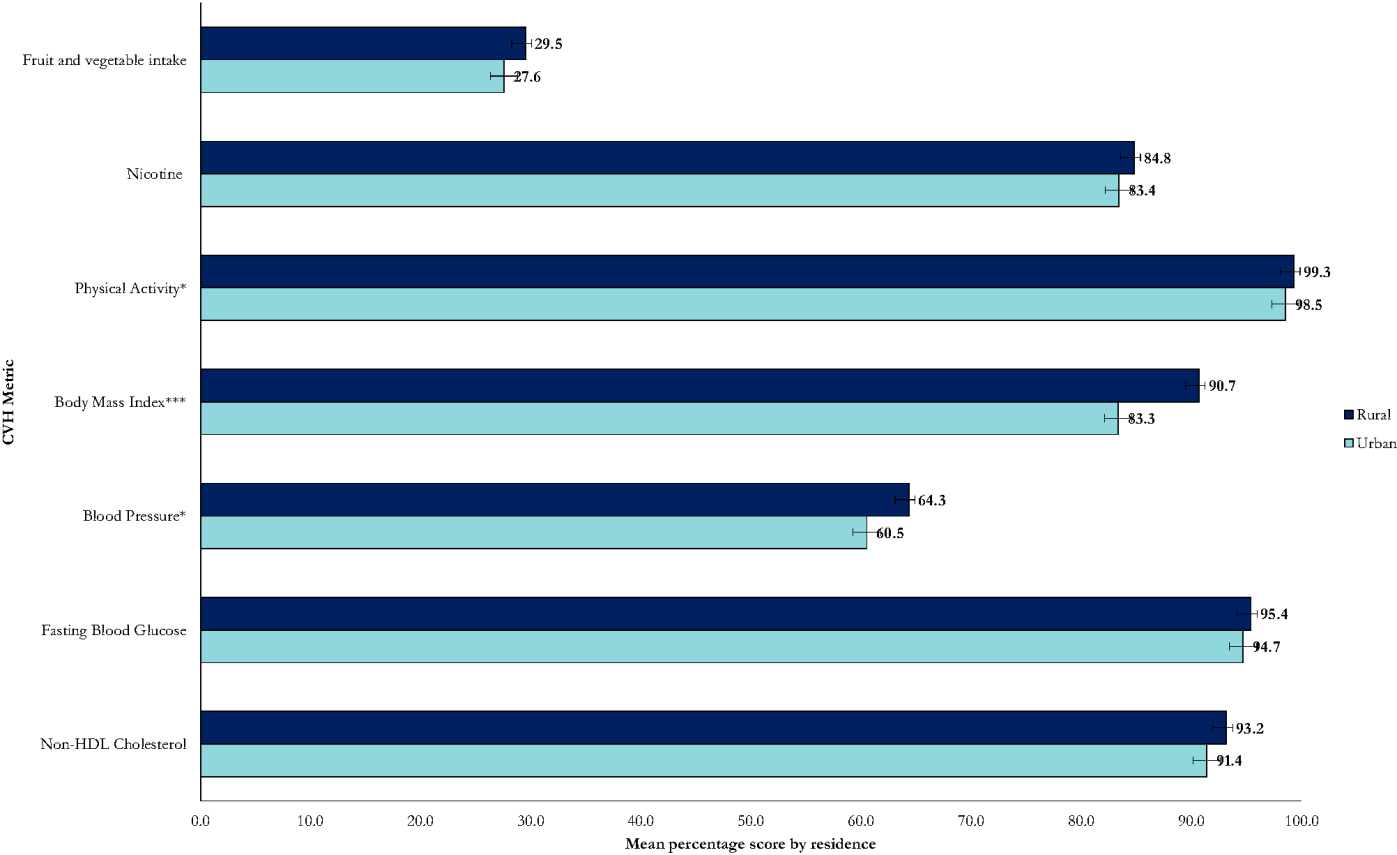
Distribution of CVH metrics by place of residence (Statistically significant difference at p < 0.001 ***, p < 0.01**, p < 0.05*).

**Figure 4:**
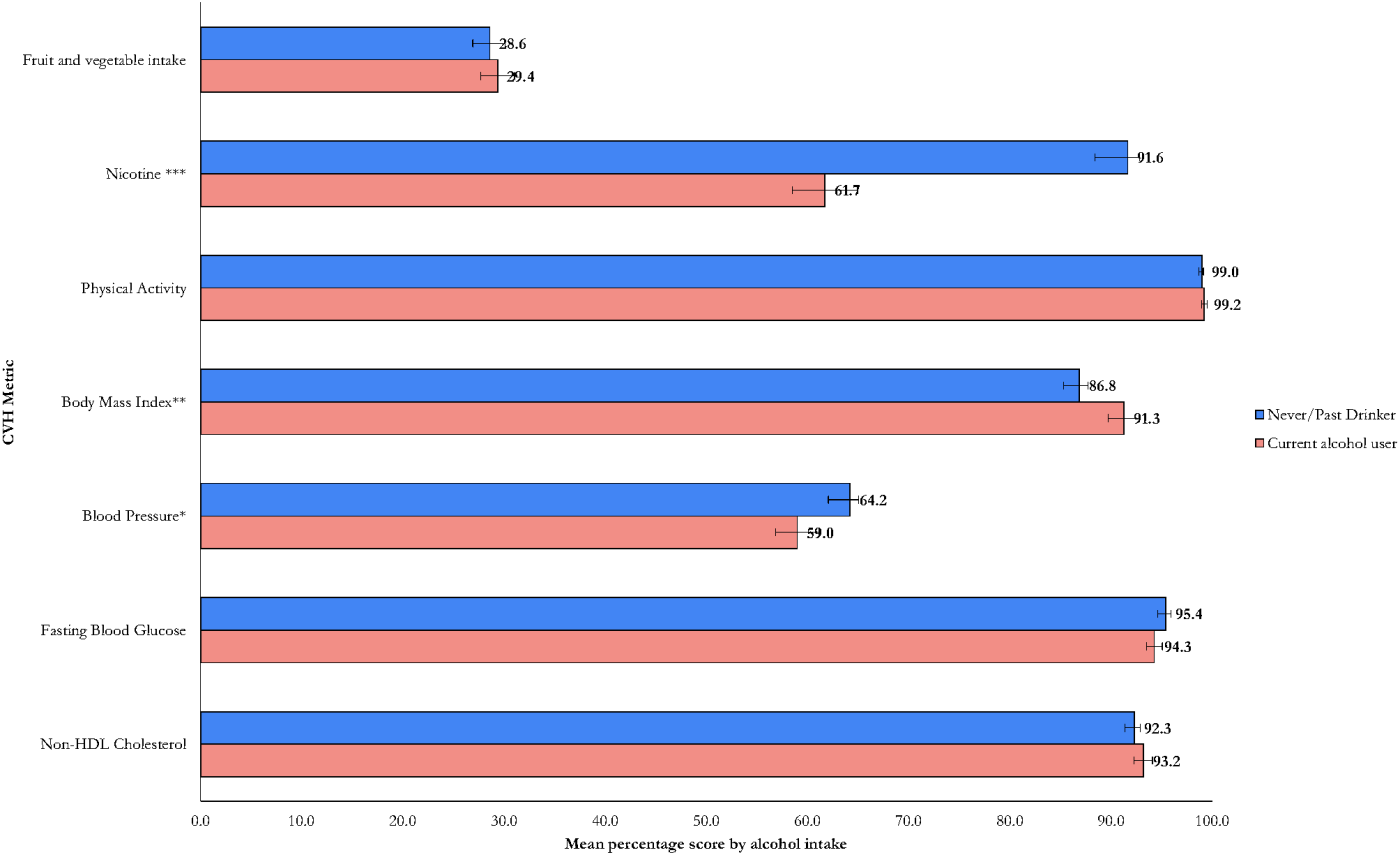
Distribution of CVH metrics by alcohol intake (Statistically significant difference at p < 0.001 ***, p < 0.01**, p < 0.05*).

The overall mean CVH score across the Kenyan population was 78.6% (95% CI: 77.9,79.2%). Figure 1 shows the shape of the distribution of the overall CVH metric. The physical activity metric had the highest overall mean score (99.0%), while fruit and vegetable intake had the lowest (28.6%). The overall mean CVH score was significantly higher in females (79.3%), people living in rural areas (79.5%), and non-drinkers of alcohol (79.6%) compared to males (77.9%), urban residents (77.0%), and alcohol drinkers (75.4%), respectively (Figures 2, 3, 4 and Table 3).

**Table 3:** Distribution of mean CVH metrics by sample characteristics.


CHARACTERISTIC	FRUIT AND VEGETABLEINTAKE	NICOTINE	PHYSICAL ACTIVITY	BODY MASS INDEX	BLOOD PRESSURE	FASTING BLOOD GLUCOSE	NON-HDL CHOLESTEROL	OVERALL CVH SCORE

	%	%	%	%	%	%	%	%

**Wealth**								

Quintile 5	20.3	75.2	99.5	95.8	69.2	94.1	93.5	78.2

Quintile 4	27.8	82.7	99.2	91.1	62.5	96.5	94.5	79.2

Quintile 3	29.0	88.0	99.3	87.2	63.3	94.8	92.2	79.1

Quintile 2	30.9	88.3	98.5	84.9	59.4	95.4	92.2	78.5

Quintile 1	35.0	86.4	98.6	80.4	59.5	94.8	90.0	77.8

**Education**								

No formal	23.7	79.0	99.1	91.4	64.9	94.7	93.3	78.0

Primary	31.3	85.8	99.1	87.2	62.0	95.8	92.9	79.2

Secondary+	31.5	88.2	98.8	84.6	61.1	94.9	91.1	78.6

**Region**								

Rift Valley	26.5	84.7	98.8	90.3	63.1	96.7	94.2	79.2

Eastern	27.4	77.4	99.0	89.2	59.9	93.8	92.0	76.9

Nyanza	45.2	93.3	99.7	88.6	68.8	96.1	91.3	83.3

Coast	27.0	75.2	98.1	88.7	64.0	93.3	93.3	77.1

Nairobi	20.5	81.6	98.9	81.6	60.2	95.6	93.8	76.0

Western	30.5	88.2	99.7	90.6	63.2	94.3	95.6	80.3

North Eastern	1.8	98.5	97.7	94.4	76.0	91.4	91.0	78.7

Central	35.4	82.4	99.3	80.8	55.8	95.4	86.7	76.5

**Ethnicity**								

Kisii	44.0	88.1	99.3	85.3	56.3	98.2	94.1	80.8

Embu	26.6	77.4	99.9	85.7	64.6	90.1	90.3	76.4

Kalenjin	25.5	89.3	99.1	88.7	61.5	96.2	92.7	79.0

Kamba	23.4	80.8	99.3	87.2	56.9	93.9	90.6	76.0

Borana	32.2	53.3	99.1	84.9	67.5	92.9	91.1	74.4

Kikuyu	35.8	81.2	98.3	82.2	59.2	95.3	92.0	77.7

Luhya	29.0	87.6	99.7	89.3	61.3	95.4	95.2	79.6

Luo	37.4	93.1	99.6	87.2	69.7	94.1	89.9	81.6

Maasai	19.2	93.2	96.5	92.2	69.8	98.8	94.5	80.6

Meru	30.2	74.2	98.9	89.6	62.2	94.7	91.5	77.3

Mijikenda	21.5	67.9	98.0	91.2	62.8	97.2	93.9	76.1

Somali	1.3	97.5	97.8	91.6	73.8	88.7	90.9	77.4

Turkana	8.9	49.0	99.1	99.6	77.7	96.4	95.0	75.1

Other	25.6	73.7	99.1	92.6	69.0	96.2	93.0	78.4


### Distribution of CVH metrics by sample characteristics

Figures 1, 2, 3, 4 and Table 3 present the distribution of the mean CVH metrics across the population.

#### CVH status by sex

Males had significantly higher mean CVH scores based on fasting blood glucose (96.3% vs. 94.0%; p < 0.01) and BMI metrics (93.1% vs. 82.7%; p < 0.001), while females had significantly higher CVH scores based on the nicotine intake (95.5% vs. 73.2%; p < 0.001). No sex differences were observed in the mean scores of fruits and vegetable intake, physical activity, blood pressure, and non-HDL cholesterol (Figure 2).

#### CVH status by place of residence

The ideal CVH prevalence was significantly higher in rural areas (48.7%) compared to urban areas (39.7%) (Table 2). Urban residents had consistently lower prevalence of ideal CVH across all the sociodemographic characteristics than rural residents. The married in urban areas had a significantly lower iCVH prevalence compared to those not in a marital union (34.5% vs. 47.1%; p = 0.002) (Table 2). There was a social gradient in the iCVH prevalence by education status among urban residents where those with at least secondary education had higher iCVH prevalence compared to those without formal education (46.3% vs. 32.7%; p = 0.021). The urban iCVH prevalence was lowest in the Central region (29.7%) while the rural prevalence was lowest in the Coast region (41.4%).

Regarding the distribution of the individual CVH metrics, rural residents had significantly higher mean CVH scores for physical activity (99.3% vs. 98.5; p < 0.05), BMI (90.7% vs. 83.3; p < 0.001), and blood pressure (64.3% vs. 60.5; p < 0.05) metrics compared to urban residents (Figure 3). The mean scores for the remaining metrics were consistently higher in rural areas but not statistically significant (Figure 3).

#### CVH status by alcohol use

Alcohol users had significantly lower scores based on nicotine (61.7% vs. 91.6; p < 0.001) and blood pressure metrics (59.0% vs. 64.2; p < 0.05), but higher scores based on BMI metric (91.3% vs. 86.8; p < 0.01) compared to non-users (Figure 4).

#### CVH status by wealth and education

Table 3 presents the distribution of CVH metrics by wealth, education, region, and ethnicity. There is no clear pattern in mean CVH scores by wealth status. The less educated individuals had significantly lower scores based on fruit and vegetable consumption and nicotine intake, but better scores based on BMI and blood pressure metrics.

#### CVH status by region and ethnicity

The Nyanza region had the highest mean scores for most metrics, while Nairobi and Central regions had the lowest overall CVH scores. The North-Eastern region had an extremely low mean score based on fruits and vegetable consumption (1.8%), but the highest scores based on nicotine (98.5%), BMI (94.4%), and blood pressure (76.0%) metrics.

Regarding ethnicity, the Kisii ethnic group had the highest fruit and vegetable consumption (44.0%) and overall CVH score (80.8%), but lowest score based on the blood pressure metric (56.3%), while the Kikuyu tribe had the lowest mean BMI scores (82.2%). Similarly, the Maasai (93.2%) and Luo (93.1%) ethnic groups had the highest scores based on nicotine metric, while the Turkana (49.0%) had the lowest.

#### Overall CVH score and 10-year CVD risk

Supplementary Figure 3 presents a scatter plot for the correlation between the overall CVH score and the predicted 10-year CVD risks based on the lab-based and non-lab-based risk CVD equations. The graph reveals a negative correlation between overall CVH score and the predicted 10-year CVD risk, a finding corroborated by Pearson’s correlation coefficient of –0.49 and -0.46 for the lab-based and non-lab-based equations, respectively.

Supplementary Figure 3: Relationship between Overall CVH Score and predicted 10-year CVD risk.

### Factors associated with ideal CVH in Kenya

Table 4 presents the results of the unadjusted and multivariable binary logistic regression analyses. The unadjusted model results show a statistically significant relationship between ideal CVH and age, marital status, occupation, alcohol intake, place of residence, region, and ethnicity. Without adjusting for other variables, the unmarried (cOR 1.2; 95% CI 1.0–1.4, p = 0.036) and Nyanza residents (cOR 1.9; 95% CI 1.2–2.9, p = 0.005) had increased odds of iCVH compared to the married and Rift valley residents, respectively. There were between 40–70% reduced odds of iCVH among the elderly compared to those below 30 years. Moreover, there were reduced odds of iCVH among urban residents (cOR 0.7; 95% CI 0.5–0.9, p = 0.004) and alcohol drinkers (cOR 0.5; 95% CI 0.4–0.6, p = 0.002), compared to rural residents and non-drinkers of alcohol. Compared to residents of the Rift valley region, residents of Eastern (cOR 0.7; 95% CI 0.5–1.0, p = 0.024) and Nairobi (cOR 0.6; 95% CI 0.4–0.9, p = 0.015) regions had reduced odds of iCVH, whereas those in the Nyanza region (cOR 1.9; 95% CI 1.2–2.9, p = 0.003) had increased odds. The unadjusted results also showed between 50–80% reduced odds of iCVH among the Kamba, Kikuyu, Luhya, Meru, Mijikenda, and Turkana ethnic groups compared to the Kisii.

**Table 4:** Factors associated with ideal CVH in Kenya (Unadjusted and Multivariable binary logistic regression analyses results for complete case analysis (n = 3818).


	COMPLETE CASE ANALYSIS (N = 3818)							

	UNADJUSTED LOGISTIC REGRESSION ANALYSIS		MULTIVARIABLE LOGISTIC REGRESSION ANALYSIS					
			
	KENYA (OVERALL)				RURAL KENYA		URBAN KENYA	
			
VARIABLE/CATEGORY	CRUDE OR (95% CI)	*P*-VALUE	ADJUSTED OR (95% CI)	*P*-VALUE	ADJUSTED OR (95% CI)	*P*-VALUE	ADJUSTED OR (95% CI)	*P*-VALUE

**Sex**								

Female	1		1		1		1	

Male	0.9 (0.7, 1.0)	0.123	1.0 (0.8, 1.3)	0.847	1.1 (0.9, 1.5)	0.373	0.9 (0.7, 1.3)	0.72

**Age group (years)**								

18–29	1		1		1		1	

30–39	**0.6 (0.4, 0.8)**	**<0.001**	**0.5 (0.4, 0.7)**	**<0.001**	**0.5 (0.4, 0.7)**	**<0.001**	0.6 (0.3, 1.1)	0.093

40–49	**0.4 (0.3, 0.6)**	**<0.001**	**0.4 (0.3, 0.5)**	**<0.001**	**0.4 (0.3, 0.6)**	**<0.001**	**0.3 (0.1, 0.6)**	**0.001**

50+	**0.3 (0.2, 0.3)**	**<0.001**	**0.2 (0.2, 0.3)**	**<0.001**	**0.2 (0.2, 0.3)**	**<0.001**	**0.3 (0.2, 0.4)**	**<0.001**

**Marital Status**								

In a union	1		1		1		1	

Not in a union	**1.2 (1.0, 1.4)**	**0.02**	1.0 (0.8, 1.2)	0.987	**0.8 (0.7, 1.0)**	**0.063**	1.3 (0.9, 2.0)	0.169

**Education**								

No formal	1		1		1		1	

Primary	1.0 (0.8, 1.3)	0.747	0.8 (0.6, 1.1)	0.172	0.8 (0.6, 1.1)	0.26	0.8 (0.4, 1.3)	0.325

Secondary +	1.3 (1.0, 1.8)	0.064	1.4 (1.0, 2.0)	0.067	1.1 (0.8, 1.6)	0.661	2.0 (1.0, 3.9)	0.055

**Occupation**								

Unemployed/Unpaid	1		1		1		1	

Self-Employed	1.3 (1.0, 1.8)	0.097	1.2 (0.9, 1.6)	0.195	1.0 (0.8, 1.4)	0.72	1.6 (0.8, 3.1)	0.146

Employed/Salaried	**1.4 (1.0, 1.9)**	**0.06**	0.9 (0.6, 1.2)	0.462	1.0 (0.6, 1.6)	0.969	0.9 (0.5, 1.6)	0.698

**Wealth quintile**								

Quintile 5	1		1		1		1	

Quintile 4	1.0 (0.8, 1.4)	0.907	0.9 (0.7, 1.2)	0.547	1.0 (0.8, 1.3)	0.954	0.8 (0.4, 1.5)	0.508

Quintile 3	1.1 (0.8, 1.5)	0.576	1.0 (0.8, 1.4)	0.83	1.2 (0.8, 1.7)	0.296	0.8 (0.5, 1.3)	0.363

Quintile 2	0.9 (0.6, 1.3)	0.573	0.9 (0.6, 1.3)	0.501	0.9 (0.6, 1.4)	0.721	0.8 (0.5, 1.4)	0.477

Quintile 1	1.1 (0.8, 1.5)	0.66	1.3 (0.9, 2.1)	0.173	1.3 (0.9, 2.0)	0.215	1.3 (0.6, 2.6)	0.481

**Alcohol intake**								

Never/Past drinker	1		1		1		1	

Current user	**0.5 (0.4, 0.6)**	**0.002**	**0.5 (0.3, 0.6)**	**<0.001**	**0.5 (0.3, 0.7)**	**<0.001**	**0.4 (0.2, 0.6)**	**<0.001**

**Residence**								

Rural	1		1		1		1	

Urban	**0.7 (0.5, 0.9)**	**0.004**	**0.6 (0.5, 0.8)**	**<0.001**	-	-	-	-

**Region**								

Rift Valley	1		1		1		1	

Eastern	**0.7 (0.5, 1.0)**	**0.024**	0.7 (0.4–1.2)	0.171	0.8 (0.3, 2.2)	0.609	0.5 (0.3, 1.0)	0.054

Nyanza	**1.9 (1.2, 2.9)**	**0.003**	1.5 (0.8–2.7)	0.176	**3.0 (1.1, 8.0)**	**0.03**	0.8 (0.4, 1.8)	0.657

Coast	0.8 (0.5, 1.2)	0.217	0.6 (0.3–1.1)	0.118	0.6 (0.2, 2.1)	0.426	**0.4 (0.2, 0.9)**	**0.025**

Nairobi	**0.6 (0.4, 0.9)**	**0.015**	**0.4 (0.2–0.8)**	**0.01**	-	-	**0.3 (0.1, 0.6)**	**0.001**

Western	1.2 (0.9, 1.6)	0.23	1.4 (0.9–1.9)	0.097	**2.1 (1.5, 2.9)**	**<0.001**	0.7 (0.4, 1.3)	0.242

North Eastern	1.1 (0.8, 1.5)	0.62	1.3 (0.4–5.1)	0.664	1.9 (0.3, 12.1)	0.483	1.1 (0.3, 4.4)	0.844

Central	0.7 (0.5, 1.0)	0.081	**0.6 (0.4–0.8)**	**0.006**	0.6 (0.4, 1.0)	0.073	**0.3 (0.2, 0.6)**	**0.001**

**Ethnicity**								

Kisii	1		1		1		1	

Embu	0.4 (0.1, 1.3)	0.116	0.8 (0.2–2.5)	0.669	1.1 (0.2–4.6)	0.947	0.8 (0.1–5.4)	0.787

Kalenjin	0.4 (0.3, 0.7)	0.002	**0.5 (0.3–0.9)**	**0.027**	0.7 (0.3–1.6)	0.394	0.5 (0.2–1.4)	0.215

Kamba	**0.3 (0.2, 0.5)**	**<0.001**	0.5 (0.2–1.2)	0.112	0.7 (0.2–2.3)	0.053	0.7 (0.2–1.8)	0.4

Borana	0.3 (0.1, 1.2)	0.084	0.4 (0.1–2.4)	0.297	-	-	0.5 (0.1–4.1)	0.477

Kikuyu	**0.5 (0.3, 0.9)**	**0.031**	0.8 (0.3–1.9)	0.64	1.2 (0.5–2.8)	0.617	0.8 (0.2–2.8)	0.729

Luhya	**0.5 (0.2, 0.9)**	**0.014**	0.5 (0.2–1.1)	0.073	0.5 (0.2–1.1)	0.09	0.7 (0.2–2.6)	0.629

Luo	0.7 (0.4, 1.3)	0.266	0.7 (0.4–1.3)	0.212	0.4 (0.2-1)	0.053	1.3 (0.5–3.2)	0.621

Maasai	0.7 (0.3, 1.4)	0.278	0.5 (0.2–1.2)	0.111	0.7 (0.3–2.1)	0.581	0.5 (0.1–2.6)	0.438

Meru	**0.5 (0.2, 0.8)**	**0.012**	0.7 (0.3–1.7)	0.489	1 (0.3–3.6)	0.949	0.5 (0.2–1.9)	0.335

Mijikenda	**0.3 (0.2, 0.8)**	**0.01**	0.5 (0.2–1.5)	0.227	0.9 (0.2–3.5)	0.85	0.7 (0.2–2.5)	0.602

Somali	0.5 (0.3, 1.0)	0.054	0.3 (0.1–1.4)	0.13	0.4 (0.1–2.4)	0.286	0.3 (0–1.8)	0.186

Turkana	**0.2 (0.1, 0.5)**	**<0.001**	**0.3 (0.1–0.6)**	**0.002**	0.3 (0.1-1)	0.054	0.4 (0.1–1.3)	0.122

Other	0.6 (0.3, 1.2)	0.164	0.9 (0.4–2.1)	0.773	1 (0.4–2.7)	0.93	1.2 (0.3–5.2)	0.82


From the adjusted model, there were between 50–80% reduced odds of ideal CVH in higher age groups compared to those aged 18–29 years. Similarly, alcohol users (AOR 0.5; 95% CI 0.3–0.6, p *<* 0.001) and urban residents (AOR 0.6; 95% CI 0.5–0.8, p *<* 0.001) had reduced odds of iCVH. Compared to residents of the Rift Valley region, residents of the Nairobi (AOR 0.4; 95% CI 0.2–0.8, p = 0.011) and Central regions (AOR 0.6; 95% CI 0.4–0.8, p = 0.006) had 40–60% reduced odds of iCVH (Table 4). Moreover, the Kalenjin (AOR 0.5; 95% CI 0.3–0.9, p *<* 0.027) and Turkana (AOR 0.3; 95% CI 0.1–0.6, p = 0.002) ethnic groups had lower odds of iCVH than the Kisii ethnic group.

The adjusted model results for rural areas are similar to the combined model except that the residents of rural Nyanza (AOR 3.0; 95% CI 1.1–8.0, p = 0.030) and rural Western (AOR 2.1; 95% CI 1.5–2.9, p *<* 0.001) regions have increased odds of having an iCVH compared to residents of rural Rift Valley. In urban settings, Coast, Nairobi, and Central regions have 60–70% reduced odds of ideal CVH compared to urban areas in the Rift valley region (Table 4).

### Model diagnostics

The Hosmer-Lemeshow test results showed a p-value of 0.2453 indicating that the model is of good fit. Similarly, the area under the ROC curve is 0.6891, indicating that the model had acceptable discriminatory power (Supplementary Figure 4). All the variance inflation factors were less than 10, with tolerance levels of above 0.1, indicating that there was no significant multicollinearity to affect the model (Supplementary Table 1).

### Model robustness and sensitivity analyses

Results from the imputed model were consistent with those of complete case analysis except for region and ethnicity (Supplementary Table 2). Compared to the Rift Valley region, Eastern, Coast, Nairobi, and Central residents have 40–50% reduced odds of iCVH. Similarly, the Kalenjin, Maasai, Somali, and Turkana had reduced odds of iCVH. The results from the adjusted ordinal logistic regression model were consistent with those of the binary model, except for region or residence, where Nyanza and North-Eastern had increased odds of iCVH (Supplementary Table 2). Additionally, the ordinal model showed that only the Somali ethnic group had a statistically significant reduced odds (AOR 0.1; 95% CI 0–0.5, p < 0.009) of iCVH compared to the Kisii.

Supplementary Table 3 presents the Bonferroni corrected results, which revealed that after adjusting for multiple comparisons, there were statistically significant reduced odds of having an ideal CVH with increasing age (p < 0.001), alcohol intake (p < 0.001), and urban residence (p = 0.005).

## Discussion

We sought to examine the prevalence of ideal CVH status in Kenyan adults and its associated sociodemographic factors. The study found a 45.6% prevalence of ideal CVH with an overall CVH score of 78.6% and poor CVH prevalence of 6.4%. Only 1.2% of Kenyan adults attained the maximum possible overall CVH score. Fruit and vegetable intake had the lowest CVH score. The prevalence of iCVH was low among males, older, urban residents, alcohol drinkers, Nairobi residents, and Turkana ethnic groups. Of all regions, residents of Nyanza had the highest prevalence of iCVH. Age, alcohol use, urban residence, ethnicity, and region of residence were associated with iCVH. In rural Kenya, marital status was associated with iCVH.

In this study, about half of Kenyan adults (45.6%) had iCVH status, attaining a mean CVH score of at least 80%. The observed prevalence is lower than the 55.9% reported in Malawi (18), 53% in rural South Africa (24), 47.6% in urban Tanzania (20), but higher than the 15.5% reported in Benin (21) and 37.4% among the urban poor in Kenya (16). Previous studies in SSA have also reported low prevalence of all the seven CVH metrics at ideal levels ranging from 3.3% in Malawi (18), 3.2% in Uganda (22), 1.2% in Benin(21), to less than 1% in rural South Africa (24), Ghana (19), and urban Tanzania (20). In Kenya, more than three-quarters of adults possess between three to six out of 12 risk factors for non-communicable diseases (10). The current prevalence of ideal CVH status in Kenya highlights the need for continued efforts to scale-up interventions aimed at promoting CVH status among the general population.

It is noteworthy that all the previous studies assessing iCVH used the 2010 AHA criteria for defining three levels for each CVH metric and hence may not accurately compare with our results. There is heterogeneity in the way previous studies have defined iCVH, with varying scales and thresholds. While some studies used 6–7 metrics (19,21,44) to define ideal CVH, others used 5–7 metrics (18,20,24). One study defined iCVH based on a score of 12–14 points on a 14 point scale (16), while another study used a five-point scale (23). The varied criteria for defining iCVH calls for the adoption of standardised approaches for assessing iCVH. The revised AHA criteria seem objective but require that studies collect systematic data on sleep health and diets. Policy makers and practitioners in the SSA region should develop or adapt the AHA metric to make it context specific and relevant to the setting for a standardised monitoring of the CVH status in SSA. This exercise would include varying the weights given to different aspects of the tool depending on the context.

Addressing the rising CVD risk factor burden is fundamental to improving the overall CVH status of the population. In this study, fruit and vegetable intake had the lowest mean score of the seven CVH metrics. This finding is consistent with previous studies in SSA, where fruit and vegetable intake had the lowest mean CVH score (18,23,24,44,45). The low rate of consumption of the recommended daily intake of fruit and vegetables could be attributed to their high cost, limited access, seasonal availability, and cultural perceptions regarding the taste of most vegetable dishes (46,47). The diet consumed affects other CVH metrics like BMI, blood pressure, sugar, and lipid levels, and is therefore quite critical to the overall CVH status (48). Relevant public health interventions are required to promote the intake of healthy diets to improve the CVH status of the Kenyan population.

The study found significant sex differences in the distribution of CVH metrics in Kenya. Females had significantly higher prevalence of ideal CVH, overall mean CVH score, and more ideal mean nicotine intake score compared to males. Previous findings in Kenya have found that males have increased odds of having multiple NCD risk factors (10) and tobacco use (7). Consistent with previous literature (29,49,50), males had significantly higher CVH scores based on BMI and blood glucose metrics compared to females. Mechanisms leading to sex differences in obesity are quite complex and could be attributed to differences in body composition, physiological processes, and lifestyles (6,51,52,53).

We found that the likelihood of iCVH decreased with advancing age. The finding mirrors those of previous studies in SSA, which have reported reduced odds of ideal CVH among the elderly (18,20,21,23). Furthermore, the prevalence of iCVH decreased with increasing age, with about six in 10 (62.4%) adults having an iCVH while only about one in four adults (25.6%) aged above 50 years having an iCVH. Ageing increases the overall risk for CVD due to the likely onset of physiological changes to the cardiovascular system associated with unhealthy lifestyles and factors. In Kenya, studies have reported increased odds of diabetes (29), hypertension (30), obesity (49), and multiple NCD risk factors (10) with increasing age. Another plausible explanation is the deterioration of lifestyle risk factors as individuals age, which reduces their likelihood of having a healthy heart. This finding highlights the need for targeted interventions aimed at improving CVH among ageing populations in Kenya. Concerted efforts are needed to diagnose and manage individuals with elevated CVD risk factors to improve the overall CVH status.

Current alcohol consumption was associated with reduced odds of iCVH status. Similarly, alcohol users had significantly lower prevalence of iCVH compared to non-users (32.3% vs. 49.6%, p < 0.001). This finding is consistent with studies conducted in similar SSA settings that have associated alcohol consumption with poor CVH (20). A plausible explanation could be that alcohol drinkers are likely to engage in other riskier lifestyles, which reduces their likelihood of ideal CVH. In our study, there was about a 30-point difference in the mean nicotine metric between alcohol users and non-users (61.6% vs. 91.6%; p < 0.001). Moreover, the mean score based on the blood pressure metric in our study was significantly lower in drinkers than non-drinkers (59.0% vs. 64.2%; p < 0.05), which could further explain this finding. It is important to scale-up campaigns against alcoholism and other drug abuse in order to improve the overall CVH of the population. Health education targeted at individuals who consume alcohol should be scaled up to promote awareness on related CVD risk factors like nicotine exposure and hypertension.

In line with previous SSA studies (18,19,21), urban residents were less likely to have an iCVH compared to rural residents. Similarly, the prevalence of iCVH was significantly lower in urban areas compared to rural areas (39.7% vs. 48.7%; p = 0.002). Our results also reveal that urban dwellers had significantly lower mean scores based on the BMI, BP, and physical activity metrics, which could explain the observed difference. Urbanisation is characterised by significant lifestyle and environmental changes leading to poor CVH status. Urban dwellers are likely to consume highly processed and poorer diets, and lead sedentary lifestyles compared to their rural counterparts. In Kenya, urban areas are characterised by higher odds of tobacco (7) and alcohol intake (54), and obesity (49) than rural areas, which could explain the observed reduced odds of iCVH among urban residents.

Compared with those residing in the Rift Valley region, Nairobi and Central region residents had reduced odds of iCVH. This finding could be attributed to the fact that both Nairobi and Central regions are more urban compared to the Rift Valley region. In this study, Nairobi had the lowest prevalence of iCVH, which can be explained by the high likelihood of unhealthy lifestyles that ultimately affect the CVH status. When only urban areas are considered, the Coast region also shows (in addition to Nairobi and Central) reduced odds of iCVH compared to the Rift Valley region. Our study shows that the Coast region has the poorest score based on the nicotine metric and has one of the lowest mean scores based on fruit and vegetable consumption and FBG metrics, which could explain the observed reduced odds of iCVH. Rural Nyanza and Western regions have increased odds of ideal CVH compared to the Rift Valley region, which can be explained by the higher mean CVH scores across all the metrics. The observed reduced odds of iCVH among the Kalenjin and Turkana ethnic groups could be explained by the considerably high mean scores across all the CVH metrics except the BP metric. Further research is required to understand the mechanisms driving ethnic variation in CVH status in Kenya.

Our study is the first to assess the ideal CVH status of Kenyan adults. The study uses the WHO STEPS dataset, which is a nationally representative dataset making our findings generalizable to Kenya. Our results are robust as confirmed by the goodness of fit (assessed by the Hosmer Lemeshow test) and other robustness tests performed. Moreover, we have performed sensitivity analyses using both binary and ordinal logistic regression models, imputed analyses, and corrected for multiple comparisons using Bonferroni correction. All the results from sensitivity analyses confirm the validity of our conclusions. To the best of our knowledge, this is the first study in SSA to use the revised AHA criteria for assessing the CVH status of the general population in the region. The findings are, therefore, important in providing the latest evidence regarding the status of iCVH status in Kenya and its drivers.

However, our study has some limitations. First, we did not include sleep health when computing the overall CVH score as recommended by the revised AHA guideline since sleep quality was not collected in the STEPS survey. Second, we only used fruit and vegetable intake as a proxy to the diet metric, which limits the scope of diet assessment. Third, there was a lack of HbA1c data, and we therefore used fasting blood glucose levels to modify the AHA criteria. Fourth, we could not infer causation due to the cross-sectional nature of the data. Nevertheless, our findings align with those from similar studies and offer initial insights into the prevalence and determinants of iCVH in Kenya.

## Conclusion

This study finds that about 45.6% of Kenyan adults have an ideal CVH, with the mean overall CVH score being 78.6% and just about one percent having a maximum CVH score. Overall, females had higher mean CVH scores and prevalence of ideal CVH compared to males. Increasing age, alcohol use, urban residence, and Nairobi, Coast and Central region residence were negatively associated with ideal CVH. The findings call for the design and scale up of specific primordial and primary CVD prevention interventions targeting individuals with different risk profiles for CVD, especially the elderly and those residing in urban areas. Policies and interventions to address harmful lifestyles like tobacco and alcohol consumption like behavioural change communication are required to improve CVH status in Kenya. Moreover, interventions targeting cardiovascular health promotion among the general population like healthy dietary practices should be ramped up. The study also highlights the need for policy makers to invest in collecting comprehensive data, especially on DASH diets and sleep practices to aid in the assessment and monitoring of the CVH status of the population. Further studies are required to explore the ethnic and geographical determinants of ideal CVH.

## Additional File

The additional file for this article can be found as follows:

10.5334/gh.1363.s1 Supplementary File. Supplementary Figures 1–3 and Tables 1–4.

## Abbreviations

AHA American Heart Association

AOR Adjusted Odds Ratio

BG Blood Glucose

BMI Body Mass Index

BP Blood Pressure

DALY Disability Adjusted Life Year

DASH Dietary Approaches to Stop Hypertension

CVD Cardiovascular Disease

CVH Cardiovascular Health

COR Crude Odds Ratio

HDL High Density Lipoprotein

iCVH Ideal Cardiovascular Health

IHD Ischemic Heart Disease

MICE Multiple Imputation with Chained Equations

NCD Non-Communicable Disease

WHO World Health Organization

ROC Receiver Operating Characteristic

SSA Sub-Saharan Africa

VIF Variance Inflation Factor
