# Supplementary material for: Prevalence and Determinants of Ideal Cardiovascular Health in Kenya: A Cross-Sectional Study Using Data From the 2015 Kenya STEPwise Survey
Source: Glob Heart. 2024 Oct 23;19(1):79. doi: 10.5334/gh.1363 (PMC11505031; doi:10.5334/gh.1363)
Supplement: Supplementary File. — Supplementary Figures 1–3 and Tables 1–4. [file gh-19-1-1363-s1.pdf]

Prevalence and Determinants of Ideal Cardiovascular Health in Kenya: A Cross-Sectional Study using Data from the 2015 Kenya STEPwise Survey

James Odhiambo Oguta[1*], Penny Breeze[1], Elvis Wambiya[1], Catherine Akoth[1], Grace Mbuthia[2], Peter Otieno[3], Oren Ombiro[4], Yvette Kisaka[5], Lilian Mbau[6], Elizabeth Onyango[5], Gladwell Gathecha[5] and Pete Dodd[1].

1. Sheffield Centre for Health and Related Research, Division of Population Health, School of Medicine and Population Health, University of Sheffield. Sheffield, S1 4DA, United Kingdom
2. School of Nursing, College of Health Sciences, Jomo Kenyatta University of Agriculture and Technology, P.O. Box 62000- 0200 Nairobi, Kenya
3. African Population and Health Research Center P.O. Box: 10787-00100, Nairobi, Kenya.
4. Medtronic LABS, Nairobi, Kenya
5. Non-Communicable Diseases Division, Ministry of Health, Afya House, Cathedral Road, Nairobi, Kenya
6. Kenya Cardiac Society, Nairobi, Kenya

Corresponding Author*- James Odhiambo Oguta; Email: mcogutajamo@gmail.com

Supplementary files

Supplementary Figure 1: Plot for the patterns of data missingness

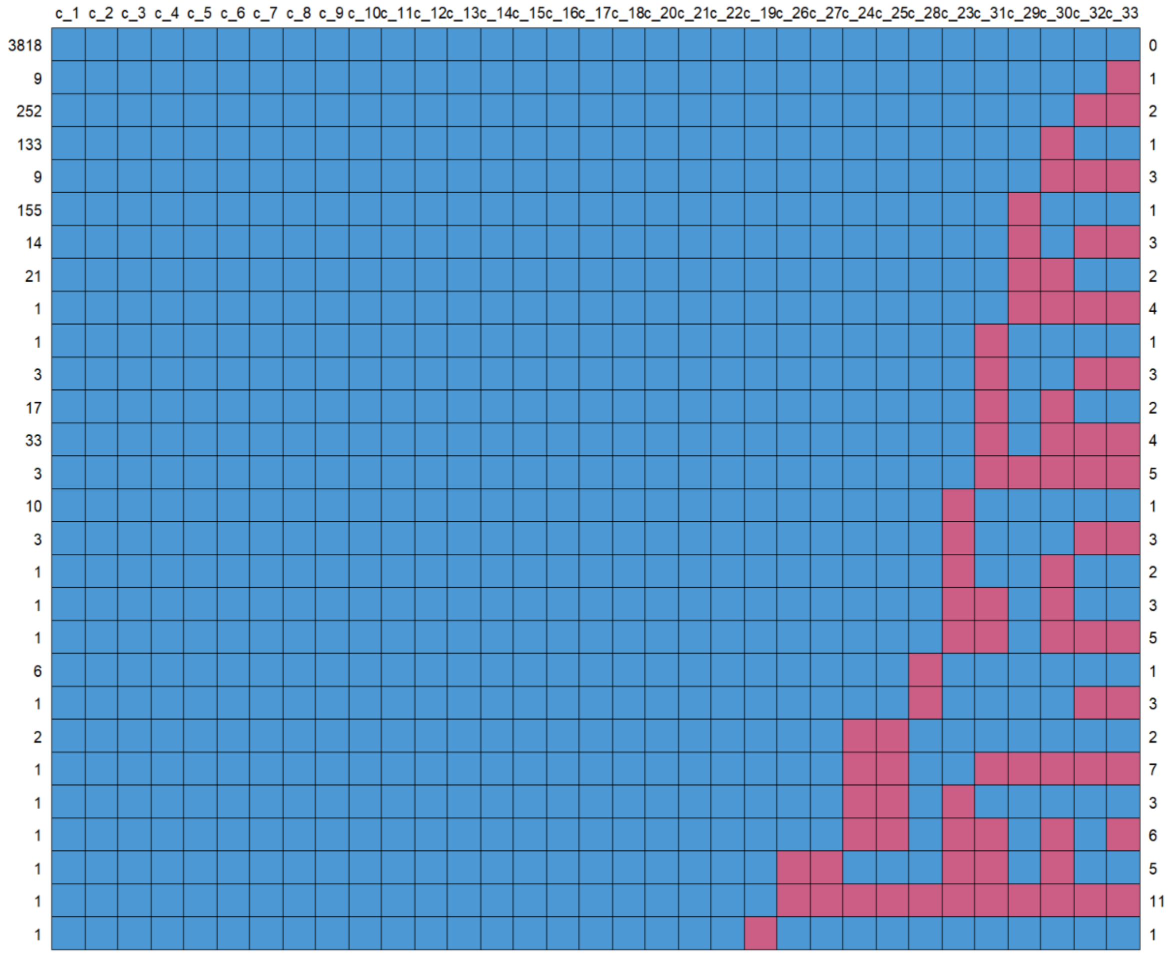

The top x axis contains the list of the variables. At the left is a count of the total number of variables for each combination with the count to the right

Supplementary Figure 2: Assessing for model convergence

Supplementary Figure 2a: Convergence plots after 20 imputations

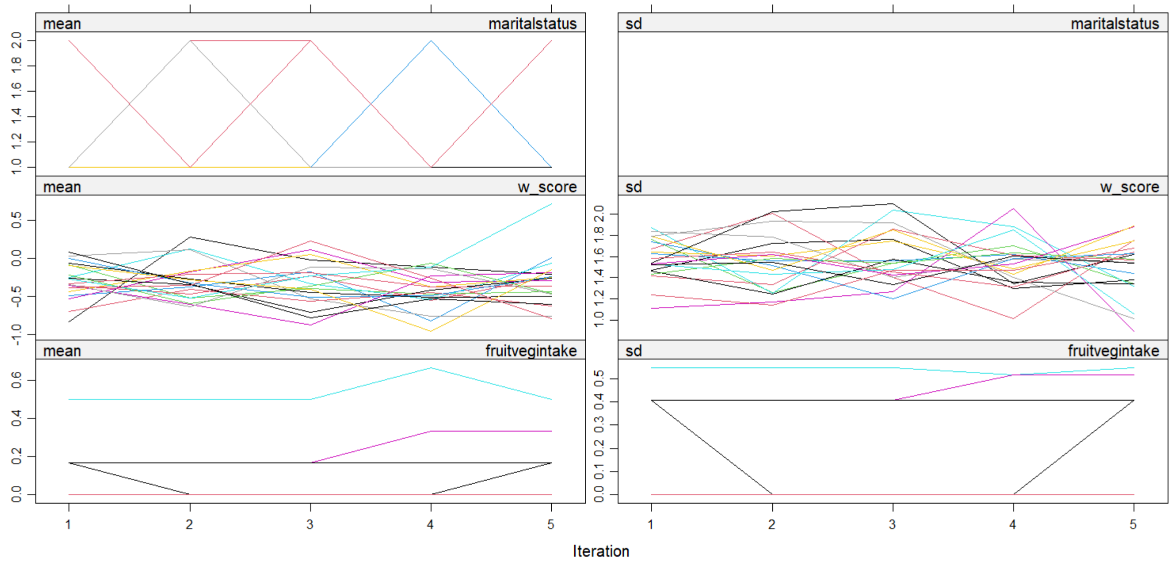

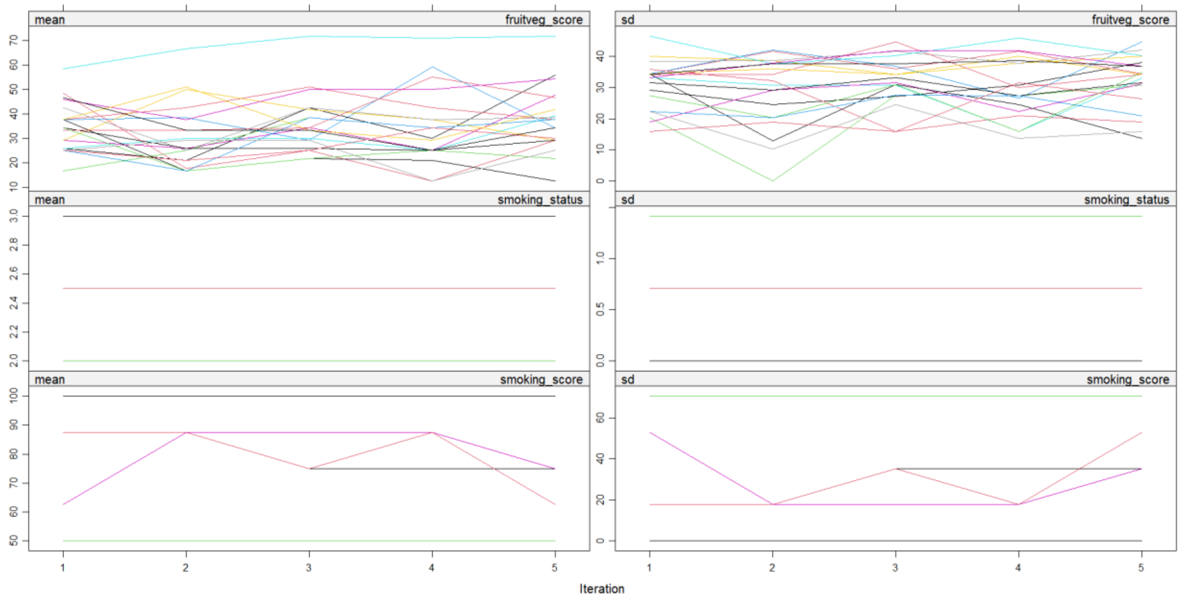

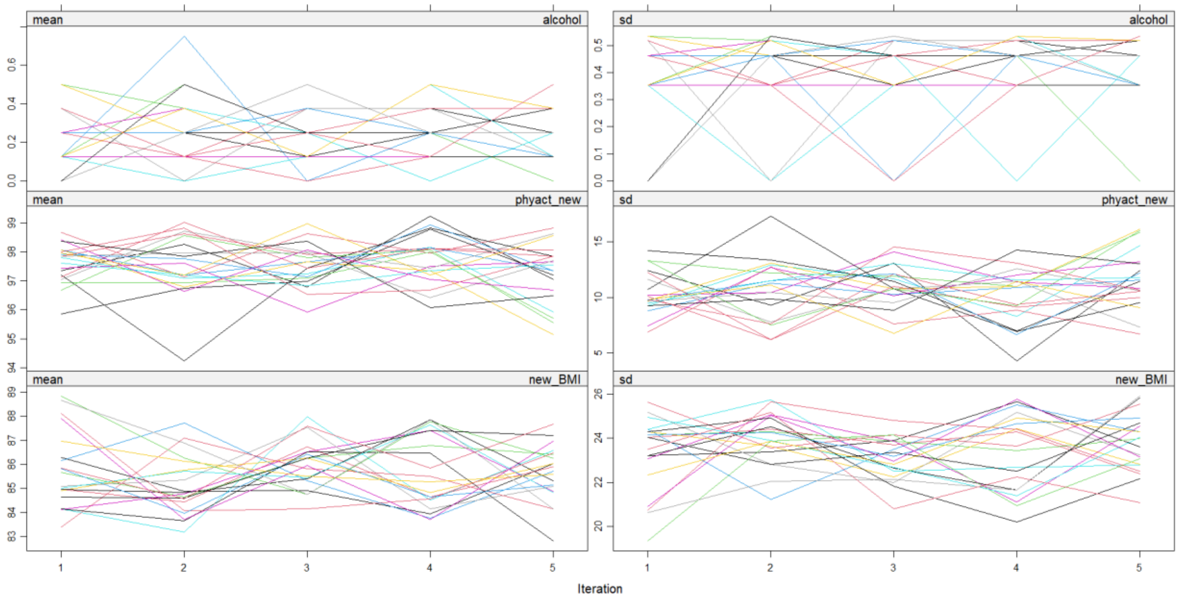
mean
alcohol
sd
alcohol
mean
phyact_new
sd
phyact_new
mean
new_BMI
sd
new_BMI
Iteration

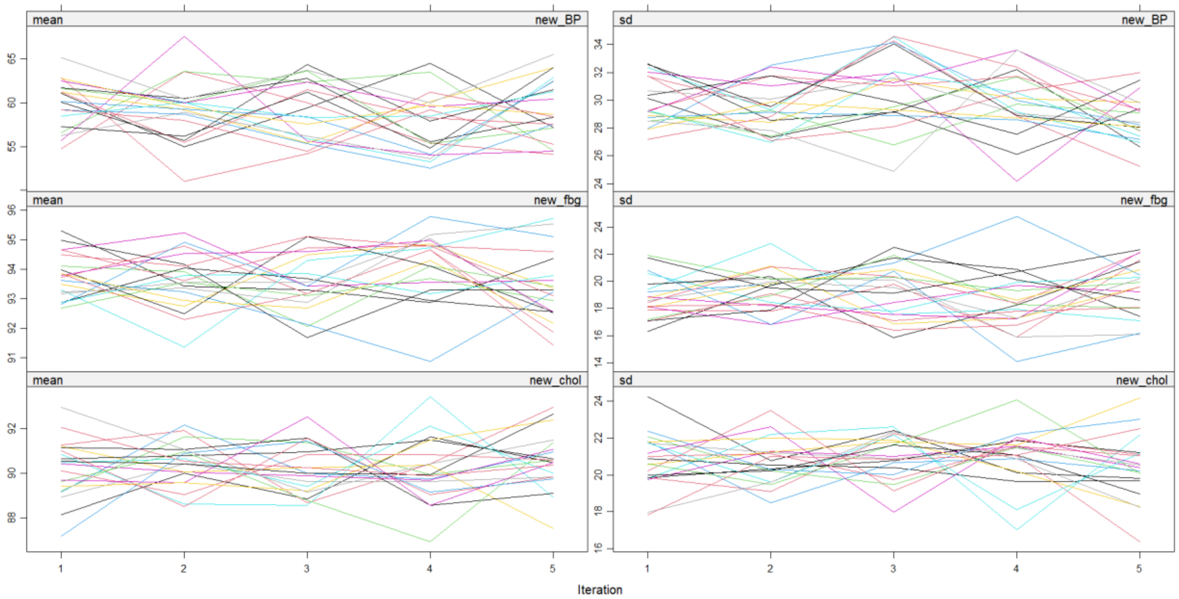
mean
new_BP
sd
new_BP
mean
new_fbg
sd
new_fbg
mean
new_chol
sd
new_chol
Iteration

Supplementary Figure 2b: Convergence plots after 80 imputations

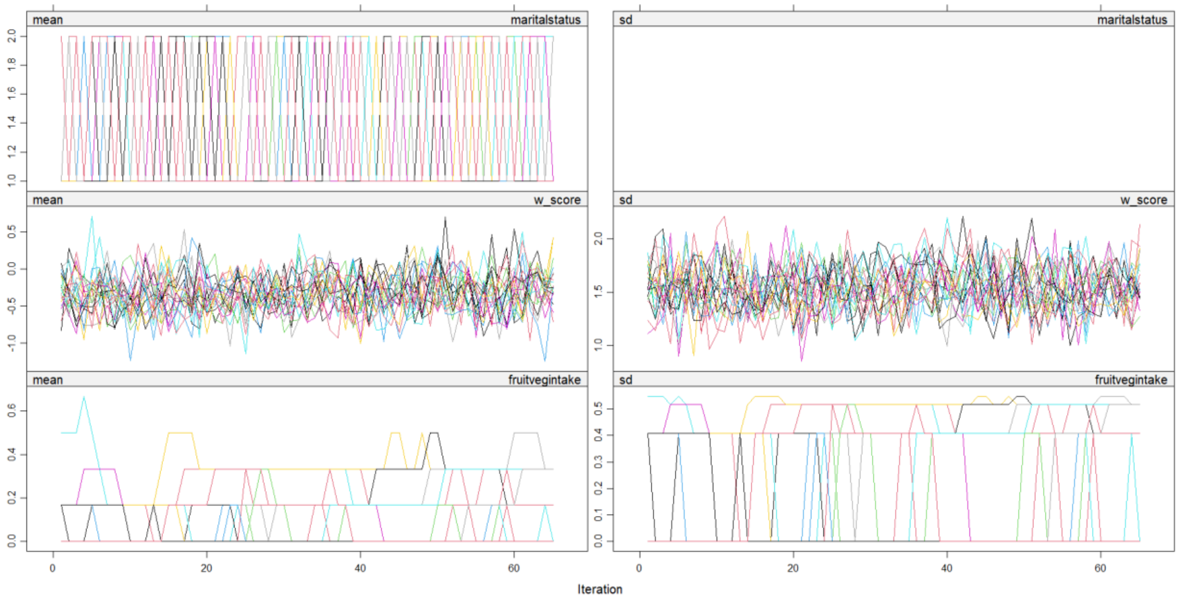

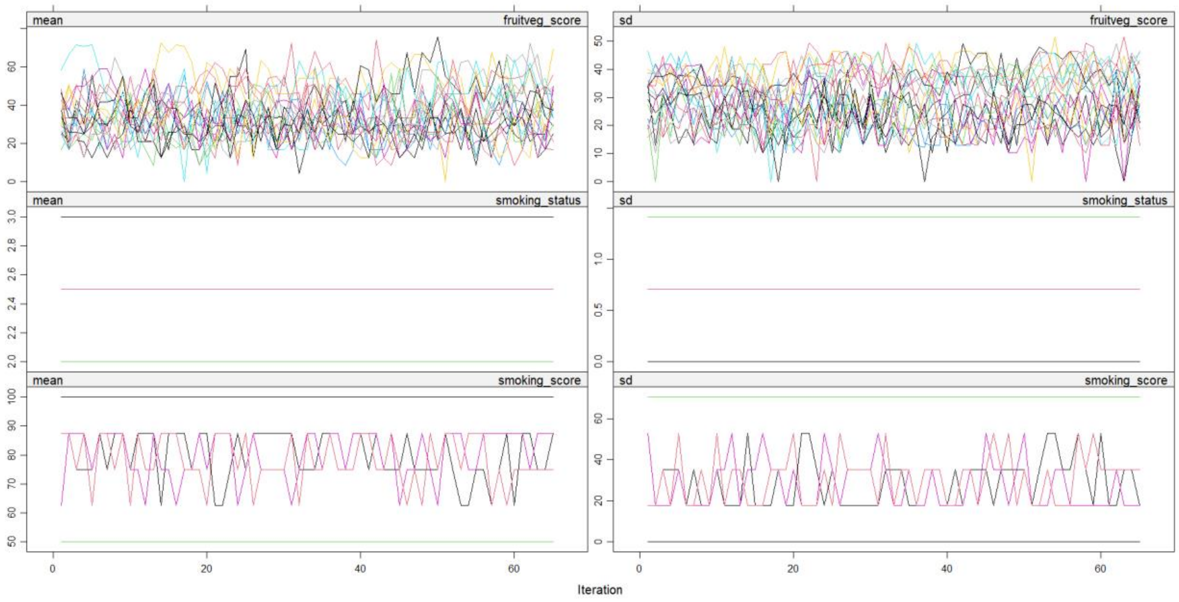

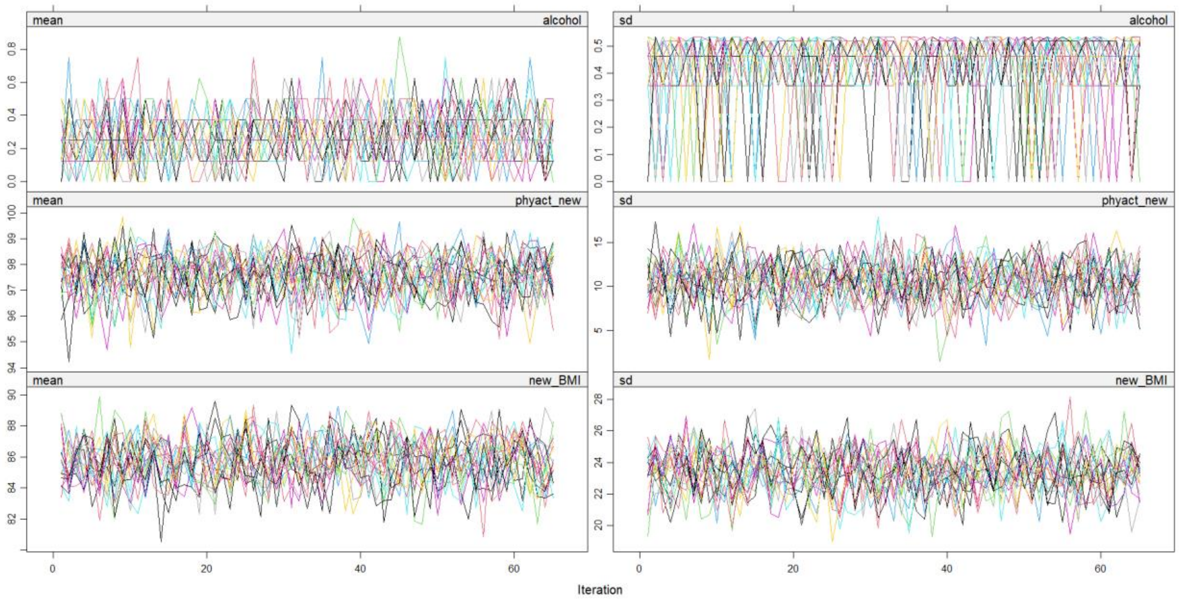
mean
alcohol
sd
alcohol
mean
phyact_new
sd
phyact_new
mean
new_BMI
sd
new_BMI
Iteration

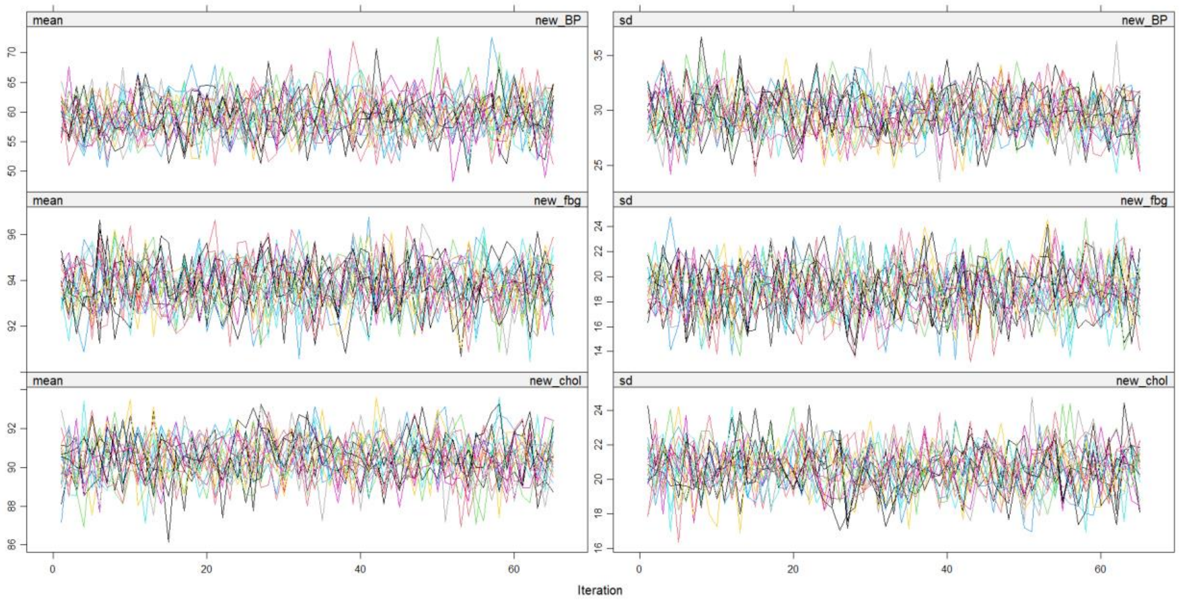
mean
new_BP
sd
new_BP
mean
new_fbg
sd
new_fbg
mean
new_chol
sd
new_chol
Iteration

Supplementary Figure 3: Relationship between Overall CVH Score and predicted 10-year CVD risk

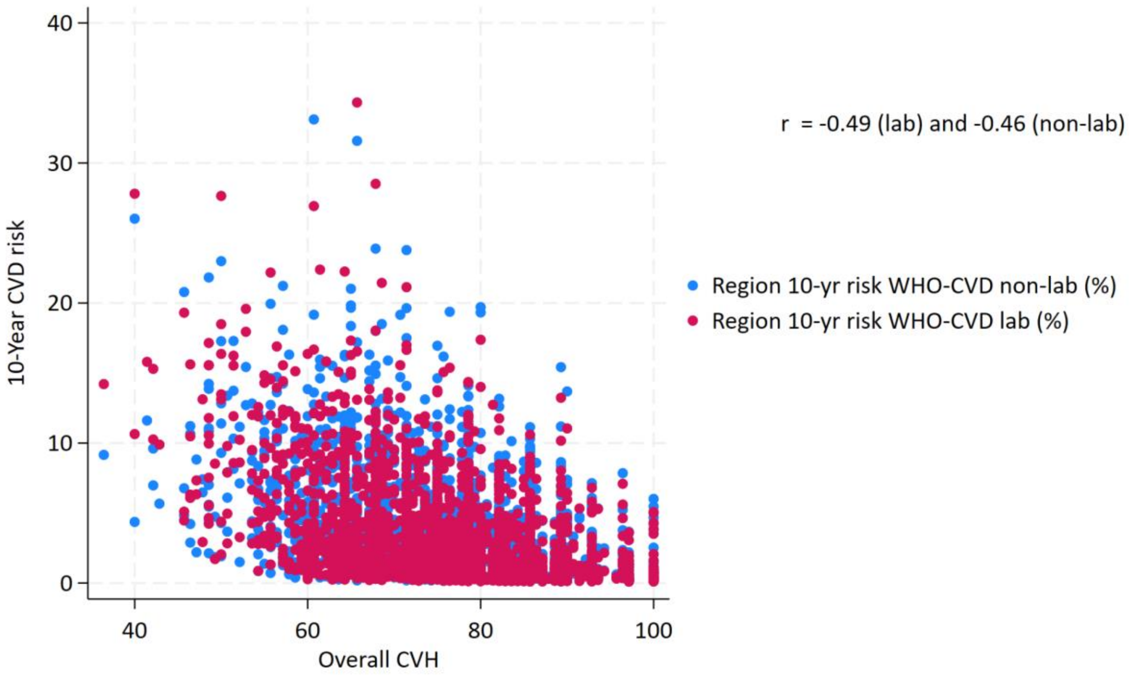

N.B- The dots represent individuals in the dataset

Supplementary Figure 4: Diagnosing model goodness of fit (ROC curve and AUC)

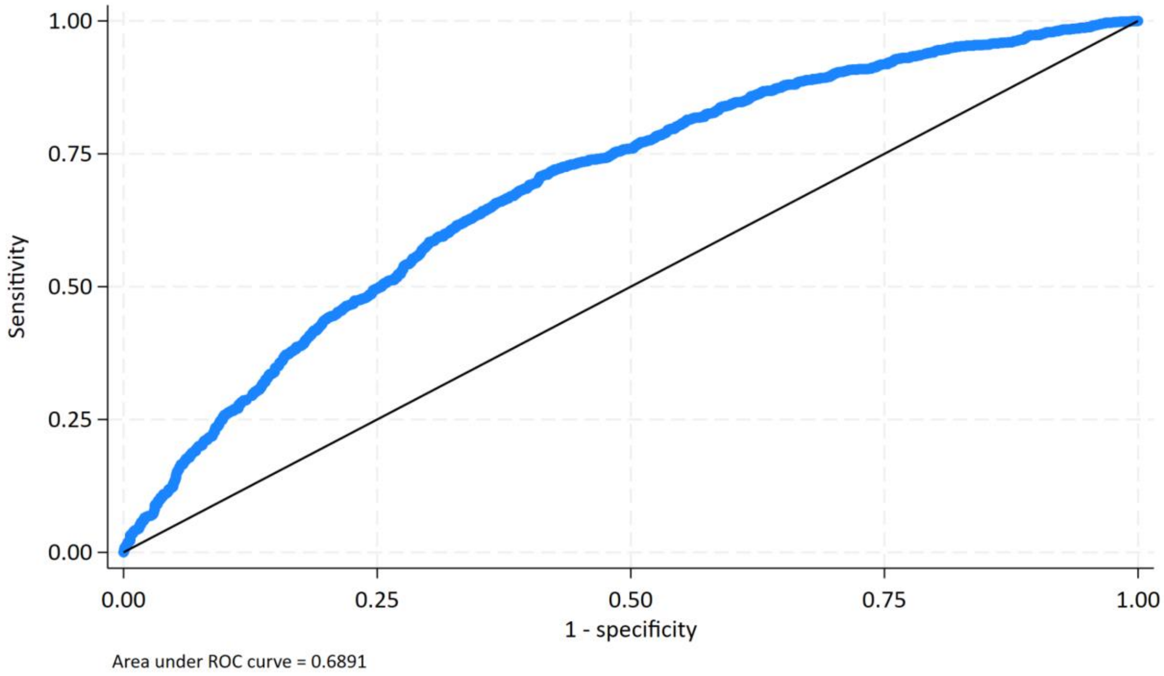

Supplementary Table 1: Assessing for Multicollinearity using variance inflation factors and tolerance

Variable/Category	VIF	Tolerance (1/VIF)
**Sex**		
Female		
Male	2.2	0.46
**Age group (years)**		
<30		
30-39	1.97	0.51
40-49	1.63	0.61
50+	1.76	0.57
**Marital Status**		
Married		
Single	1.58	0.63
**Education**		
No formal		
Primary	2.22	0.45
Secondary +	2.75	0.36
**Occupation**		
Unemployed/Unpaid		
Self-Employed	2.33	0.43
Employed/Salaried	1.97	0.51
**Wealth quintile**		
Quintile 5		
Quintile 4	2.2	0.45
Quintile 3	2.3	0.43
Quintile 2	3	0.40
Quintile 1	2.79	0.36
**Alcohol intake**		
Never/Past drinker		
Current user	1.55	0.65
**Residence**		
Rural		
Urban	2.31	0.43
**Region**		
Rift Valley		
Eastern	4.86	0.21
Nyanza	2.65	0.38
Coast	2.96	0.34
Nairobi	1.1	0.91

Western	2.59	0.39
North Eastern	9.47	0.11
Central	2.34	0.43
**Ethnicity**		
Kisii		
Embu	1.66	0.60
Kalenjin	2.19	0.46
Kamba	3.47	0.29
Borana	1.16	0.86
Kikuyu	3.61	0.28
Luhya	3.4	0.29
Luo	2.56	0.39
Maasai	1.09	0.91
Meru	2.76	0.36
Mijikenda	2.03	0.49
Somali	9.62	0.10
Turkana	1.12	0.89
Other	2.21	0.45
**Mean VIF**	**2.71**	

VIF- Variance inflation factor

Supplementary Table 2: Sensitivity analysis from imputed dataset using multiple imputation (n=4500)

	**Imputed model results-Sensitivity analyses**			
	**Binary logistic Regression Model**		**Ordinal logistic Regression Model**	
**Variable/Category**	**Adjusted OR (95% CI)**	***P*-Value**	**Adjusted OR (95% CI)**	***P*-Value**
**Sex**				
Female	1			
Male	1 (0.9 - 1.3)	0.932	0.9 (0.8 - 1.1)	0.327
**Age group (years)**				
>30	1			
30-39	**0.5 (0.4 - 0.7)**	**<0.001**	**0.5 (0.4 - 0.6)**	**<0.001**
40-49	**0.4 (0.3 - 0.5)**	**<0.001**	**0.4 (0.3 - 0.5)**	**<0.001**
50+	**0.2 (0.2 - 0.3)**	**<0.001**	**0.2 (0.1 - 0.2)**	**<0.001**
**Marital Status**				
In a union	1			
Not in a union	1 (0.9 - 1.2)	0.888	1 (0.9 - 1.1)	0.936
**Education**				
No formal	1			
Primary	0.8 (0.7 - 1.1)	0.153	1 (0.8 - 1.2)	0.713

Secondary +	1.2 (0.9 - 1.7)	0.186	1 (0.8 - 1.3)	0.726
**Occupation**				
Unemployed/Unpaid	1			
Self-Employed	1.1 (0.8 - 1.4)	0.545	1 (0.8 - 1.2)	0.884
Employed/Salaried	0.8 (0.6 - 1.1)	0.176	0.9 (0.7 - 1.2)	0.491
**Wealth quintile**				
Quintile 5	1			
Quintile 4	0.9 (0.7 - 1.2)	0.526	1 (0.8 - 1.4)	0.911
Quintile 3	1 (0.8 - 1.4)	0.847	1 (0.8 - 1.3)	0.945
Quintile 2	0.9 (0.6 - 1.2)	0.352	1 (0.8 - 1.3)	0.99
Quintile 1	1.3 (0.9 - 2)	0.14	1.3 (1 - 1.7)	0.09
**Alcohol intake**				
Never/Past drinker	1			
Current user	**0.4 (0.3 - 0.6)**	**<0.001**	**0.4 (0.3 - 0.5)**	**<0.001**
**Residence**				
Rural	1			
Urban	**0.7 (0.6 - 0.9)**	**0.001**	**0.7 (0.6 - 0.8)**	**<0.001**
**Region**				
Rift Valley	1			

Eastern	**0.6 (0.4 - 0.9)**	**0.029**	0.8 (0.5 - 1.2)	0.318
Nyanza	1.7 (1 - 3)	0.069	**2.7 (1.6 - 4.5)**	**<0.001**
Coast	**0.6 (0.4 - 0.9)**	**0.015**	0.8 (0.6 - 1.2)	0.345
Nairobi	**0.5 (0.3 - 1)**	**0.045**	0.6 (0.4 - 0.8)	0.004
Western	1.2 (0.7 - 1.9)	0.591	1.1 (0.7 - 1.6)	0.786
North Eastern	1.2 (0.5 - 2.7)	0.674	**8.8 (1.2 - 65.6)**	**0.033**
Central	**0.6 (0.4 - 0.9)**	**0.016**	0.7 (0.5 - 1.1)	0.143
**Ethnicity**				
Kisii	1		1	
Embu	0.9 (0.3 - 2.9)	0.89	1 (0.4 - 2.5)	0.978
Kalenjin	**0.5 (0.3 - 0.9)**	**0.029**	1 (0.7 - 1.7)	0.834
Kamba	0.7 (0.3 - 1.4)	0.275	0.8 (0.4 - 1.4)	0.394
Borana	0.5 (0.1 - 2.2)	0.375	0.5 (0.1 - 2.4)	0.384
Kikuyu	0.7 (0.3 - 1.6)	0.46	0.9 (0.5 - 1.9)	0.877
Luhya	0.7 (0.3 - 1.5)	0.336	1.4 (0.7 - 2.6)	0.338
Luo	0.6 (0.4 - 1.1)	0.12	1.1 (0.6 - 1.9)	0.724
Maasai	**0.5 (0.2 - 0.9)**	**0.034**	0.8 (0.4 - 1.3)	0.293
Meru	0.9 (0.4 - 2.1)	0.868	1 (0.5 - 1.9)	0.938
Mijikenda	0.7 (0.3 - 1.7)	0.399	0.6 (0.3 - 1.3)	0.206

Somali	**0.3 (0.1 - 0.9)**	**0.027**	**0.1 (0 - 0.5)**	**0.009**
Turkana	**0.2 (0.1 - 0.6)**	**0.002**	0.6 (0.3 - 1.3)	0.183
Other	0.8 (0.4 - 1.7)	0.632	1.1 (0.5 - 2.3)	0.808

Bold is statistically significant at p<0.05

Supplementary Table 3: Adjusting for multiple comparisons using Bonferroni correction

	**Multivariable binary logistic regression analysis**		
**Variable/Category**	**Adjusted OR (95% CI)**	***Original P-*** **Value**	**Bonferroni corrected P-Value**
**Sex**			
Female	1		
Male	1.0 (0.8, 1.3)	0.847	1.000
**Age group (years)**			
18-29	1		
30-39	**0.5 (0.4, 0.7)**	**<0.001**	**0.001**
40-49	**0.4 (0.3, 0.5)**	**<0.001**	**<0.001**
50+	**0.2 (0.2, 0.3)**	**<0.001**	**<0.001**
**Marital Status**			
Married	1		
Single	1.0 (0.8, 1.2)	0.987	1.000
**Education**			
No formal	1		
Primary	0.8 (0.6, 1.1)	0.172	1.000
Secondary +	1.4 (1.0, 2.0)	0.067	1.000
**Occupation**			
Unemployed/Unpaid	1		
Self-Employed	1.2 (0.9, 1.6)	0.195	1.000
Employed/Salaried	0.9 (0.6, 1.2)	0.462	1.000
**Wealth quintile**			
Quintile 5	1		
Quintile 4	0.9 (0.7, 1.2)	0.547	1.000
Quintile 3	1.0 (0.8, 1.4)	0.83	1.000
Quintile 2	0.9 (0.6, 1.3)	0.501	1.000
Quintile 1	1.3 (0.9, 2.1)	0.173	1.000
**Alcohol intake**			
Never/Past drinker	1		
Current user	**0.5 (0.3, 0.6)**	**<0.001**	**<0.001**

**Residence**			
Rural	1		
Urban	**0.6 (0.5, 0.8)**	**<0.001**	**0.005**
**Region**			
Rift Valley	1		
Eastern	0.7 (0.4 - 1.2)	0.171	1.000
Nyanza	1.5 (0.8 - 2.7)	0.176	1.000
Coast	0.6 (0.3 - 1.1)	0.118	1.000
Nairobi	**0.4 (0.2 - 0.8)**	**0.01**	0.466
Western	1.4 (0.9 - 1.9)	0.097	1.000
North Eastern	1.3 (0.4 - 5.1)	0.664	1.000
Central	**0.6 (0.4 - 0.8)**	**0.006**	0.281
**Ethnicity**			
Kisii	1		
Embu	0.8 (0.2 - 2.5)	0.669	1.000
Kalenjin	**0.5 (0.3 - 0.9)**	**0.027**	1.000
Kamba	0.5 (0.2 - 1.2)	0.112	1.000
Borana	0.4 (0.1 - 2.4)	0.297	1.000
Kikuyu	0.8 (0.3 - 1.9)	0.64	1.000
Luhya	0.5 (0.2 - 1.1)	0.073	1.000
Luo	0.7 (0.4 - 1.3)	0.212	1.000
Maasai	0.5 (0.2 - 1.2)	0.111	1.000
Meru	0.7 (0.3 - 1.7)	0.489	1.000
Mijikenda	0.5 (0.2 - 1.5)	0.227	1.000
Somali	0.3 (0.1 - 1.4)	0.13	1.000
Turkana	**0.3 (0.1 - 0.6)**	**0.002**	0.110
Other	0.9 (0.4 - 2.1)	0.773	1.000

Bold is statistically significant at $p<0.05$

Supplementary Table 4: STROBE Statement—Checklist of items that should be included in reports of *cross-sectional studies*

	Item No	Recommendation	Page No
**Title and abstract**	1	(*a*) Indicate the study's design with a commonly used term in the title or the abstract	1
		(*b*) Provide in the abstract an informative and balanced summary of what was done and what was found	2
**Introduction**			
Background/rationale	2	Explain the scientific background and rationale for the investigation being reported	3-4
Objectives	3	State specific objectives, including any prespecified hypotheses	4
**Methods**			
Study design	4	Present key elements of study design early in the paper	4
Setting	5	Describe the setting, locations, and relevant dates, including periods of recruitment, exposure, follow-up, and data collection	4
Participants	6	(*a*) Give the eligibility criteria, and the sources and methods of selection of participants	4
Variables	7	Clearly define all outcomes, exposures, predictors, potential confounders, and effect modifiers. Give diagnostic criteria, if applicable	5
Data sources/ measurement	8*	For each variable of interest, give sources of data and details of methods of assessment (measurement). Describe comparability of assessment methods if there is more than one group	*5*
Bias	9	Describe any efforts to address potential sources of bias	N/A
Study size	10	Explain how the study size was arrived at	N/A
Quantitative variables	11	Explain how quantitative variables were handled in the analyses. If applicable, describe which groupings were chosen and why	6-7
Statistical methods	12	(*a*) Describe all statistical methods, including those used to control for confounding	6-7
		(*b*) Describe any methods used to examine subgroups and interactions	6-7
		(*c*) Explain how missing data were addressed	6-7
		(*d*) If applicable, describe analytical methods taking account of sampling strategy	6-7
		(*e*) Describe any sensitivity analyses	6-7
**Results**			
Participants	13*	(a) Report numbers of individuals at each stage of study—eg numbers potentially eligible, examined for eligibility, confirmed eligible, included in the study, completing follow-up, and analysed	7

		(b) Give reasons for non-participation at each stage	7
		(c) Consider use of a flow diagram	N/A
Descriptive data	14*	(a) Give characteristics of study participants (eg demographic, clinical, social) and information on exposures and potential confounders	7
		(b) Indicate number of participants with missing data for each variable of interest	7
Outcome data	15*	Report numbers of outcome events or summary measures	7-10
Main results	16	(*a*) Give unadjusted estimates and, if applicable, confounder-adjusted estimates and their precision (eg, 95% confidence interval). Make clear which confounders were adjusted for and why they were included	10-11
		(*b*) Report category boundaries when continuous variables were categorized	10-11
		(*c*) If relevant, consider translating estimates of relative risk into absolute risk for a meaningful time period	N/A
Other analyses	17	Report other analyses done—eg analyses of subgroups and interactions, and sensitivity analyses	11
**Discussion**			
Key results	18	Summarise key results with reference to study objectives	12
Limitations	19	Discuss limitations of the study, taking into account sources of potential bias or imprecision. Discuss both direction and magnitude of any potential bias	15
Interpretation	20	Give a cautious overall interpretation of results considering objectives, limitations, multiplicity of analyses, results from similar studies, and other relevant evidence	12-15
Generalisability	21	Discuss the generalisability (external validity) of the study results	15
**Other information**			
Funding	22	Give the source of funding and the role of the funders for the present study and, if applicable, for the original study on which the present article is based	16

*Give information separately for exposed and unexposed groups.

Note: An Explanation and Elaboration article discusses each checklist item and gives methodological background and published examples of transparent reporting. The STROBE checklist is best used in conjunction with this article (freely available on the Web sites of PLoS Medicine at http://www.plosmedicine.org/, Annals of Internal Medicine at http://www.annals.org/, and Epidemiology at http://www.epidem.com/). Information on the STROBE Initiative is available at www.strobe-statement.org.
